# Whole‐tree nonstructural carbohydrate storage and seasonal dynamics in five temperate species

**DOI:** 10.1111/nph.15462

**Published:** 2018-10-12

**Authors:** Morgan E. Furze, Brett A. Huggett, Donald M. Aubrecht, Claire D. Stolz, Mariah S. Carbone, Andrew D. Richardson

**Affiliations:** ^1^ Department of Organismic and Evolutionary Biology Harvard University 26 Oxford St Cambridge MA 02138 USA; ^2^ Department of Biology Bates College Lewiston ME 04240 USA; ^3^ Center for Ecosystem Science and Society Northern Arizona University Flagstaff AZ 86011 USA; ^4^ Department of Biological Sciences Northern Arizona University Flagstaff AZ 86011 USA; ^5^ School of Informatics, Computing, and Cyber Systems Northern Arizona University Flagstaff AZ 86011 USA

**Keywords:** carbon allocation, carbon balance, Harvard Forest, nonstructural carbohydrates (NSCs), temperate trees, whole‐tree NSC storage

## Abstract

- Despite the importance of nonstructural carbohydrates (NSC) for growth and survival in woody plants, we know little about whole‐tree NSC storage. The conventional theory suggests that NSC reserves will increase over the growing season and decrease over the dormant season. Here, we compare storage in five temperate tree species to determine the size and seasonal fluctuation of whole‐tree total NSC pools as well as the contribution of individual organs.
- NSC concentrations in the branches, stemwood, and roots of 24 trees were measured across 12 months. We then scaled up concentrations to the whole‐tree and ecosystem levels using allometric equations and forest stand inventory data.
- While whole‐tree total NSC pools followed the conventional theory, sugar pools peaked in the dormant season and starch pools in the growing season. Seasonal depletion of total NSCs was minimal at the whole‐tree level, but substantial at the organ level, particularly in branches. Surprisingly, roots were not the major storage organ as branches stored comparable amounts of starch throughout the year, and root reserves were not used to support springtime growth.
- Scaling up NSC concentrations to the ecosystem level, we find that commonly used, process‐based ecosystem and land surface models all overpredict NSC storage.

## Introduction

Existing primarily as nonstructural carbohydrates (NSCs), and to a lesser degree as lipids and sugar alcohols, nonstructural carbon (C) plays a critical role in the physiology and metabolism of forest trees. NSCs are stored in essentially all living vegetative tissues in the form of soluble sugars and insoluble starch and can be subsequently drawn upon to maintain proper tree function. They serve as building blocks for growth, fuel for respiration, and solutes for osmoregulation and osmoprotection (reviewed in Hartmann & Trumbore, 2016). As such, NSCs are allocated to various functions and stored in various organs on timescales spanning minutes to decades, allowing trees to persist when respiration exceeds photosynthesis during recurring annual events like springtime leaf out in deciduous species as well as during more unpredictable stressors like drought.

As trees rely on and replenish stored NSCs throughout the year, seasonal variation in storage is driven by the balance between sources and sinks. Depletion occurs when photosynthesis is low or demands are high, and refilling occurs under the reverse conditions (Chapin *et al*., 1990). Based on the conventional theory of annual NSC reserve dynamics in temperate forest woody plants, NSCs are expected to increase throughout the growing season when photosynthesis is high and NSC reserves accumulate as growth slows, and decrease throughout the dormant season when photosynthesis is absent and NSC reserves are drawn upon for respiration (Kozlowski, 1992). However, our understanding of NSC storage – at the whole‐tree level – remains limited. Specifically, we lack a detailed understanding of how the size and seasonal fluctuation of whole‐tree total NSC storage as well as the contributions of individual organs to these dynamics differ among temperate forest trees.

While previous studies have estimated total NSC storage at the whole‐tree level (Gholz & Cropper, 1991; Barbaroux *et al*., 2003; Hoch *et al*., 2003; Würth *et al*., 2005; Gough *et al*., 2009; Richardson *et al*., 2015; Smith *et al*., 2017), whole‐tree total NSC storage has not been assessed throughout the year with high temporal resolution. Generating estimates of whole‐tree total NSC storage requires a detailed assessment in which NSC concentrations are frequently measured across organs, scaled up to the whole‐organ level, and then summed together. These estimates are essential for understanding how C flows throughout trees over time and will help to interpret NSC dynamics as a product of the complex integration of source‐sink functions, storage strategies, and different biological roles of sugars and starch (reviewed in Martínez‐Vilalta *et al*., 2016).

Moreover, previous work supports the need to measure a wider range of both belowground and aboveground organs, as a single organ is not a good indicator of NSC storage at the whole‐tree level (Richardson *et al*., 2013). NSC concentrations differ between organs, making it impossible to estimate whole‐tree storage based on concentration measurements from a single organ. Also, the seasonal dynamics may be different for each organ (Hoch *et al*., 2003), reflecting an organ's contribution to physiological and metabolic processes at different times throughout the year as well as its physiological specialization. For example, roots may have a higher storage capacity due to a larger proportion of ray and axial parenchyma cells (Lens *et al*., 2000; Pratt *et al*., 2007), with NSC reserves allocated towards new springtime growth. Thus, some organs may preferentially store NSCs more so than other organs, with this role shifting seasonally.

In addition to contributions by individual organs, a species’ ecology also drives the seasonal dynamics of whole‐tree total NSC storage. Differences in NSC storage and allocation are influenced by leaf habit (Hoch *et al*., 2003; Palacio *et al*., 2007; Richardson *et al*., 2015) and wood anatomy (Barbaroux & Bréda, 2002). For example, past studies often report higher storage requirements for deciduous than evergreen species (Dickson, 1989; Kozlowski, 1992; Hoch *et al*., 2003). Similar differences in storage are also evident based on wood anatomy, with larger reserves for ring‐porous compared to diffuse‐porous species (Barbaroux & Bréda, 2002). These findings motivate the estimation of whole‐tree total NSC storage across multiple species, which in turn fosters a more robust estimation of total NSC storage at the ecosystem level.

Furthermore, our understanding of the seasonal dynamics of whole‐tree total NSC storage is limited (Würth *et al*., 2005; Smith *et al*., 2017). While total NSC concentrations at the organ level have been found to be only weakly seasonal (Hoch *et al*., 2003; Richardson *et al*., 2013; Hoch, 2015), a detailed within‐year study is needed to assess seasonality at the whole‐tree level. By examining whole‐tree total NSC pools during periods of varying supply and demand over the course of a year, we can resolve the contribution of individual organs, as well as characterize seasonal fluctuations of sugar and starch pools to determine both the size and timing of annual minima/maxima. Although not yet quantified, a minimum threshold of NSC storage may be required to maintain proper tree function (Adams *et al*., 2013). Thus, a detailed within‐year study not only provides foundational insights into the role of NSCs in whole‐tree and ecosystem C balance, but also informs future studies that seek to investigate the influence of interannual variation and associated stressors (i.e. drought) on storage dynamics.

**Figure 1:**
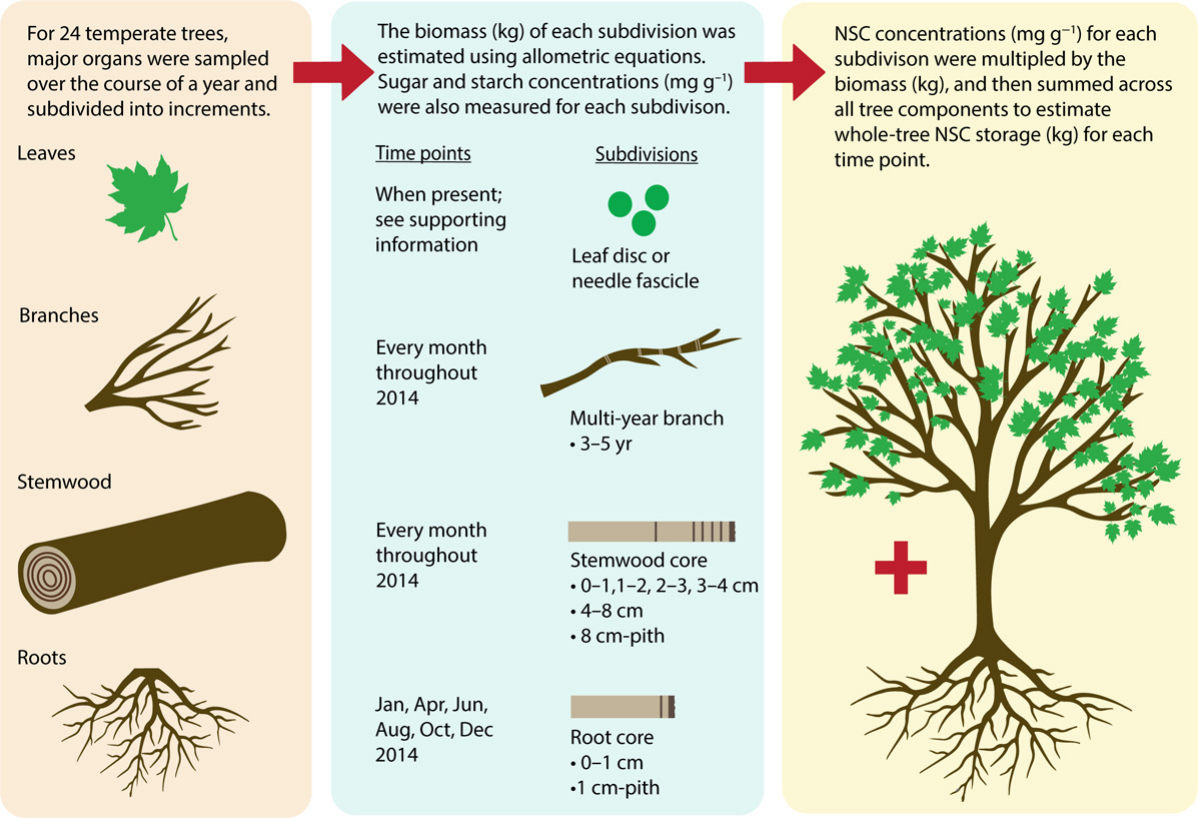
Summary of field sampling and allometric scaling from nonstructural carbohydrate (NSC) concentrations to whole‐tree NSC pools.

Here we characterize whole‐tree total NSC storage in five temperate tree species. We collected belowground and aboveground woody organs each month, measured their sugar and starch concentrations, and then scaled these data up to the whole‐tree level (Fig. 1). Our objective was to quantify whole‐tree total NSC storage over the course of a year to test the conventional theory, which suggests that NSC reserves will increase over the growing season and decrease over the dormant season. Specifically, we addressed the following questions: (1) How big are whole‐tree total NSC pools? (2) Do these pools vary across the seasons and if so, what is the degree of seasonal fluctuation? (3) What is the contribution of individual organs to whole‐tree storage? (4) Do the above storage dynamics differ between coexisting temperate tree species? Additionally, to understand the role of storage in the context of ecosystem‐level C fluxes and annual woody biomass production, we estimated ecosystem‐level total NSC storage using forest stand inventory data and compared this with predictions from a suite of commonly used, process‐based ecosystem and land surface models.

## Materials and Methods

### Study site

Harvard Forest, is an oak‐dominated, mixed temperate forest located in Petersham, MA, USA (42.53°N, 72.17°W). We selected 24 mature trees for this study belonging to the following species: red oak (*Quercus rubra* L., *n *=* *6), white pine (*Pinus strobus* L., *n *=* *6), red maple (*Acer rubrum*,* n *=* *6), paper birch (*Betula papyrifera*,* n* = 3), and white ash (*Fraxinus americana* L., *n *=* *3). These trees represent the dominant species in this area of Harvard Forest and are broadly representative of the forests in the northeastern USA. Importantly, they cover various forms of leaf habit, wood anatomy, and shade tolerance. White pine is an evergreen conifer, whereas the other species are deciduous broadleaf trees. Of the deciduous broadleaf species, red oak and white ash are ring‐porous, and red maple and paper birch are diffuse‐porous. White ash and paper birch are shade‐intolerant species, while the others are of intermediate shade tolerance.

Throughout the study year 2014, phenological observations (O'Keefe, 2015) as well as environmental conditions (Boose, 2018) were recorded at Harvard Forest and provide context for NSC dynamics reported herein. In brief, our study species exhibited 50% budburst by mid‐May. In the autumn, leaves began to drop by the start of October and deciduous species were barren by mid‐November. A comprehensive analysis of phenological events across tree species and over time at Harvard Forest is provided in Richardson & O'Keefe (2009). Furthermore, Harvard Forest has a mean annual temperature of 7.1°C and a mean annual precipitation of 1100 mm. Air temperature and precipitation data recorded by an on‐site meteorological station in 2014 as well as for the period 2002–2017 are displayed in Supporting Information Fig. S1.

### Field collection

In January 2014, we measured diameter at breast height (DBH) and tree height (Table  S1) for all sampled trees. Each month throughout 2014, a stemwood core to the pith was collected from each tree with a standard 4.3‐mm increment borer (Haglöf Company Group, Långsele, Sweden), starting at breast height on the south or southwest face of each tree with each subsequent core collected in a zigzag pattern (*c*. 7.5 cm over, 7.5 cm up; 18 cm average core depth). In addition to a monthly stemwood core, we collected sunlit branches from the top of the canopy, which was accessed using a bucket lift. Sunlit leaves were also gathered when present, but were not necessarily taken from the sampled branch. We collected coarse root cores in January, April, June, August, October, and December 2014. The first root sample was taken at 20 cm along the root from the base of the tree, and subsequent cores were taken in a zigzag pattern. Samples were kept on dry ice in the field during each collection and then stored at −80°C.

### Laboratory preparations and NSC analyses

It has been shown that NSC concentrations often decline with increasing stem depth (Hoch *et al*., 2003), yielding NSC concentrations in the heartwood that are generally very low. Therefore, scaling to the whole‐stem requires that this variation be accounted for. Although other work has scaled up by differentiating between sapwood and heartwood (Würth *et al*., 2005), we subdivided the stemwood cores into smaller pieces to obtain a finer resolution. Coarse roots were also subdivided, and branchwood was homogenized across multiple years of growth. Therefore, across trees, we consistently subdivided organs for NSC analysis (Methods S1).

Importantly, NSCs were measured in both ‘inactive’ and ‘active’ tissues due to our approach of subdividing entire organs. Given the diameter of the sampled roots (*c*. ≥ 5 cm diameter) and branches (*c*. 1–1.5 cm diameter, multi‐year 3–5 yr old), ‘inactive’ heartwood may have been present in the roots, but perhaps absent or minimal in the branches depending on the species. Less than 20% of NSCs in the stem and 10% of NSCs in the whole tree were stored in the stem heartwood. Inclusion of the heartwood in pool estimates did not affect the seasonal dynamics of NSC storage in the stem (Methods S2).

Samples were freeze‐dried (FreeZone 2.5; Labconco, Kansas City, MO, USA, and Hybrid Vacuum Pump, Vacuubrand, Wertheim, Germany) and ground (mesh 20, Thomas Scientific Wiley Mill, Swedesboro, NJ, USA; SPEX SamplePrep 1600; MiniG, Metuchen, NJ, USA). To measure sugar concentrations (adapted from Chow & Landhäusser, 2004), 10 mg of previously freeze‐dried and ground tissue was freeze‐dried overnight and then extracted with 80% hot ethanol followed by colorimetric analysis with phenol−sulfuric acid. The resulting bulk sugar extract was read at 490 nm on a microplate reader (Epoch Microplate Spectrophotometer; Bio‐Tek Instruments, Winooski, VT, USA) or a spectrophotometer (Thermo Fisher Scientific GENESYS 10S UV‐Vis, Waltham, MA, USA). Sugar concentrations (expressed as mg sugar per g dry wood) were calculated from a 1 : 1 : 1 glucose−fructose−galactose (Sigma Chemicals, St Louis, MO, USA) standard curve.

To determine starch concentrations, the remaining tissue was solubilized in NaOH and then digested with an α‐amylase/amyloglucosidase digestive enzyme solution. Glucose hydrolysate was determined using a PGO‐colour reagent solution (Sigma Chemicals) and read at 525 nm. Starch concentrations (expressed as mg starch per g dry wood) were calculated based on a glucose (Sigma Chemicals) standard curve. When conducting NSC analyses, we included at least one internal laboratory standard (red oak stemwood, Harvard Forest, Petersham, MA, USA) per analysis. Additional information about NSC measurements are provided in Methods S1.

### Allometric scaling from sugar and starch concentrations to whole‐organ and whole‐tree pools

We estimated the dry wood biomass of each organ (branch, stemwood, and root; Table  S2) and organ subdivisions using allometric scaling theory (Jenkins *et al*., 2004; see Methods S2 for details of calculations). The species‐specific allometric equations used were developed for North American trees > 2.5 cm DBH, which made them appropriate for the species and size range of trees in this study. To reiterate, subdividing organs allowed us to account for variation in NSC concentrations within an organ (i.e. radial decline of NSCs in stemwood). We then paired the sugar and starch concentrations for each sample (i.e. subdivisions of each organ) with the estimate of that component's woody biomass. This was done for each sample per tree, and then the amounts were summed to estimate whole‐tree total NSC storage for each month. In this case, the whole‐tree total NSC pool is the sum of coarse root, stemwood, and branch reserves.

Jenkins *et al*. (2004) does not provide equations and coefficients for distinguishing between coarse and fine roots. Thus, only coarse roots were sampled and coarse root biomass was estimated in this study (Methods S2). A lack of partitioning of roots into different diameter size classes may ultimately lead to underestimation or overestimation of NSC storage in the root system. Uncertainty estimates for fine roots are provided in Richardson *et al*. (2015).

**Figure 2:**
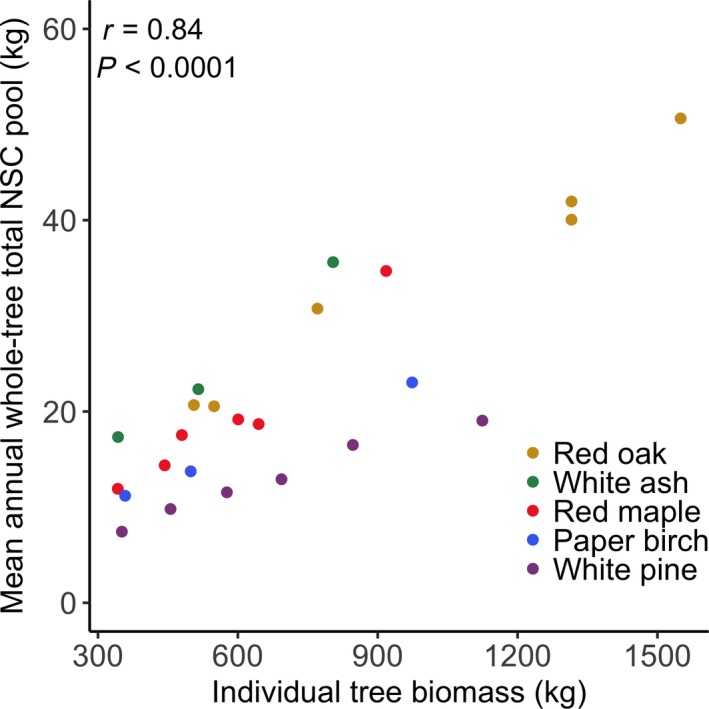
Relationship between tree biomass and mean annual whole‐tree total nonstructural carbohydrate (NSC) pools for 24 trees sampled at Harvard Forest in 2014. Strength of association was evaluated using Pearson's correlation, α = 0.05.

For a subset of trees, we also included the NSC pool in foliage, but our calculations indicated that this was a minor portion of the whole‐tree total NSC pool (Methods S3). Additionally, trees in our sample were all of similar DBH and ultimately biomass based on allometric equations. Therefore, biomass did not significantly differ between species (Fig.  S2) and storage differences reflect NSC concentrations. However, there was a positive correlation between individual tree biomass and whole‐tree total NSC pool size (Fig. 2), so biomass was included as a covariate in our statistical analyses. Data from this project are available for download and public use (Furze *et al*., 2018).

### Estimating ecosystem‐level total NSC storage

In 2014, a biomass inventory of the Prospect Hill Tract at Harvard Forest was conducted for live trees > 10 cm DBH in 34, 10 m radius plots (Munger & Wofsy, 2018). Using these data and species‐specific allometric equations, we estimated the biomass of each tree and its organs (foliage, branch, stemwood, and root) for over 600 trees from 14 deciduous broadleaf and evergreen conifer species (Methods S4). Measured total NSC concentrations from our five study species were used as estimates for the most similar tree species and paired with woody biomass to obtain whole‐tree total NSC storage. We then summed together whole‐tree total NSC storage of individual trees to estimate total NSC storage per unit ground area for this temperate forest, and examined this value in the context of annual woody biomass production, eddy flux tower measurements, and model‐based predictions of total NSC storage at Harvard Forest. See caption of Fig. 6 for process‐based model assumptions. When comparing results, assume that NSCs are *c*. 40% C to convert from kg NSC m^2^ to kg C m^2^.

### Statistical analyses

While stemwood and branches were sampled every month, roots were sampled in January, April, June, August, October, and December 2014. Therefore, statistical analyses were conducted for these 6 months when whole‐tree total NSC, sugar, and starch pools were complete and represent the sum of root, stem, and branch reserves. Statistical analyses for organ‐level dynamics used 6‐month data for roots and 12‐month data for stemwood and branches. All statistical analyses were performed in R v.3.3.2 and linear mixed‐effects (lme) models were fit by maximum‐likelihood using the nlme package. All models contain fixed effects (specified below), individual tree as a random effect, and whole‐tree or whole‐organ biomass as a covariate. For significant mixed‐effects models, differences between pairs of means were evaluated with Tukey's honest significant difference (HSD), α = 0.05.

**Figure 3:**
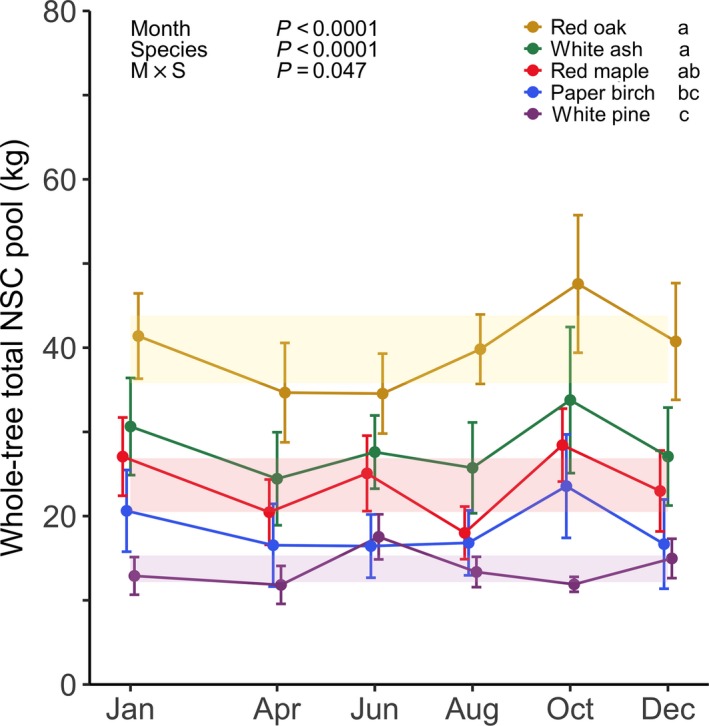
Seasonal dynamics of whole‐tree total nonstructural carbohydrate (NSC) pools for five temperate tree species sampled at Harvard Forest in 2014. Error bars denote ± 1 SE of the mean. Lowercase letters indicate significance of differences among species. Shaded bands for individual species represent the 95% confidence interval around the linear mixed‐effects (lme) model estimated mean whole‐tree total NSC pool.

**Figure 4:**
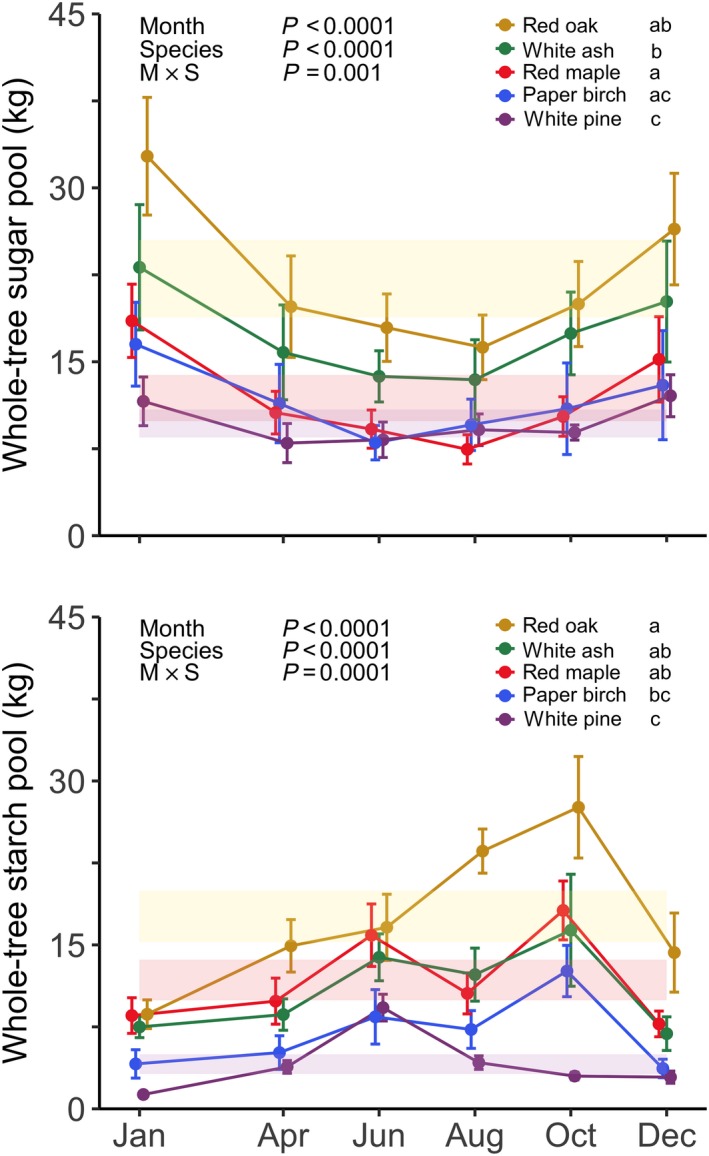
Seasonal dynamics of whole‐tree sugar (top) and starch (bottom) pools for five temperate tree species sampled at Harvard Forest in 2014. Error bars denote ± 1 SE of the mean. Lowercase letters indicate significance of differences among species. Shaded bands for individual species represent the 95% confidence interval around the lme model estimated mean whole‐tree sugar or starch pool.

To compare whole‐tree total NSC pool size between our five temperate species, as well as to determine if whole‐tree total NSC pools varied across seasons according to the conventional theory, we used a linear mixed‐effects (lme) model to analyze whole‐tree total NSC (sum of sugar and starch pools) pool size among sampling months and species (month × species; Fig. 3). The same analysis was repeated for both whole‐tree sugar and starch pools (month × species; Fig. 4). For significant interaction effects, whole‐tree total NSC, sugar, and starch pools were assessed across sampling months for each individual species (month; shaded bands in Figs 3, 4; Table S3).

**Figure 5:**
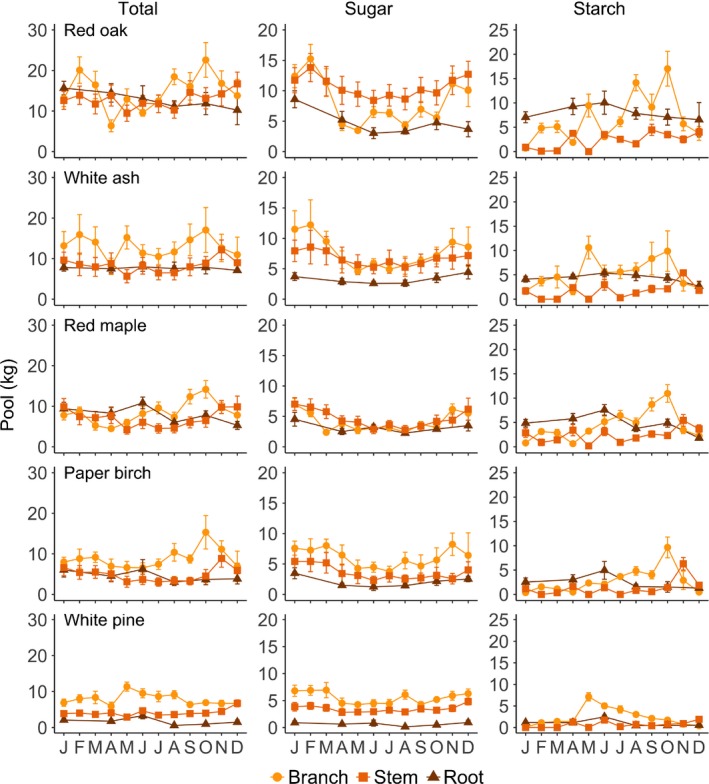
Seasonal dynamics of whole‐organ total nonstructural carbohydrate (NSC) (left), sugar (middle), and starch (right) pools for five temperate tree species (rows) sampled monthly at Harvard Forest in 2014. Error bars denote ± 1 SE of the mean. Note the difference in *y*‐axis scale between columns.

Next, we sought to determine if pool size and seasonal patterns differed at the organ level by assessing total NSC, sugar, and starch pools in the branches, stemwood, and roots (Fig. 5). For each pool type, we used a lme model to analyze pool size among organs and species (organ × species, Table  S4). For significant interaction effects, total NSC, sugar, and starch pools were assessed among organs for each individual species (organ; Table  S5). Finally, to determine if organ‐level storage varied across the seasons, we used a lme model to analyze total NSC, sugar, and starch pools for each organ among sampling months and species (month × species; Table  S6). Again, for significant interaction effects, each pool type was assessed across sampling months for each individual species and organ (month; Table  S7).

## Results

### Whole‐tree total NSC pools

The ranking of species from largest to smallest whole‐tree total NSC pool was red oak, white ash, red maple, paper birch, and white pine, and pool size significantly differed between species (*P *<* *0.0001; Fig. 3). These results support the idea that species fall along a gradient according to leaf habit and wood anatomy, with deciduous ring‐porous species (red oak and white ash) having larger whole‐tree total NSC reserves than both deciduous diffuse‐porous (red maple and paper birch) and evergreen conifer (white pine) species.

In general, whole‐tree total NSC pools built up over the growing season and declined over the dormant season, which is in line with the conventional theory (*P *<* *0.0001; Fig. 3). However, the effect of sampling month on whole‐tree total NSC pools depended on species (*P *=* *0.047). The whole‐tree total NSC pool tended to peak in October for each deciduous species and in June for evergreen white pine (Table  S3). This finding suggests that leaf habit may also influence seasonal dynamics as whole‐tree total NSC pools peak at different times of the year for deciduous and evergreen species. However, while the timing of peak storage at the whole‐tree level may differ between species, the magnitude of seasonal NSC fluctuation was similar. During the growing season, deciduous species exhibited an *c*. 28% increase in whole‐tree total NSC pools from the mean minimum in April to the mean maximum in October. This increase was comparable for white pine from April to June (32%).

### Whole‐tree sugar and starch pools

Whole‐tree sugar and starch pools differed between species (both *P *<* *0.0001; Fig. 4). Whole‐tree sugar pools were largest for the ring‐porous species red oak and white ash. In most cases, whole‐tree starch pools for deciduous species were larger than that of white pine. In general, NSCs were more often stored as sugars rather than starch throughout the year, but most deciduous species retained substantial reserves in the form of starch by the end of the growing season.

In general, whole‐tree sugar pools decreased over the first half of the year and replenished during the second half of the year, peaking in the winter; whereas whole‐tree starch pools slowly increased throughout the year reaching a peak in October (both *P *<* *0.0001; Fig. 4). However, the effect of sampling month on whole‐tree sugar and starch pools depended on species (sugar *P *=* *0.001; starch *P *=* *0.0001). Whole‐tree sugar pools followed the expected pattern for all species, whereas whole‐tree starch pools tracked the patterns for total NSC pools, specifically the peak in October for the deciduous species and in June for evergreen white pine (Fig. 4; Table S3).

### Storage and seasonality in organs

The distribution of total NSCs, sugars, and starch differed between organs (all *P *<* *0.0001; Fig. 5; Table S4). Despite stemwood having the largest biomass, branches were the largest reservoir of total NSCs and sugars, followed by roots, and then stemwood. For starch, branches and roots had comparable storage which was larger than that of stemwood. However, the partitioning between organs depended on species (Table  S4), and starch storage did not always differ between organs, particularly for paper birch (Table  S5).

The seasonal patterns of sugar and starch pools (Table  S6) often differed between organs, possibly reflecting different functional roles of sugars and starch in each organ and/or different organ contributions to processes occurring at different times of the year. In general, sugar pools were lower in the growing season than in the dormant season, particularly in aboveground organs. By contrast, while branch and stem starch pools tended to peak at the onset of the dormant season in deciduous species, root starch, and root total NSC pools in general, remained fairly stable throughout the year. However, root total NSC and starch pools declined between June and August for red maple, and root sugar pools declined between January and April for red oak and red maple (Table  S7).

The percentage of sugars and starch in each organ relative to whole‐tree total NSCs varied throughout the year (Table  S8). As noted above, whole‐tree total NSC pools tended to peak in October for each deciduous species and in June for white pine. At these times of maximal storage, organ contributions to the whole‐tree total NSC pool were *c*. 25% root, 25% stemwood, and 50% branch for deciduous species and 20% root, 25% stem, and 55% branch for white pine. However, the distribution of sugars and starch differed across these woody organs. Between 70% and 90% of whole‐tree sugar was in aboveground organs. The majority of whole‐tree starch was stored in the branches of deciduous species in October (red oak 63%, white ash 57%, red maple 60%, and paper birch 77%), with far less in the roots (*c*. 30% in red oak, white ash, and red maple, and 13% in paper birch).

### Estimation of ecosystem‐level total NSC storage and seasonality

We estimated the belowground and aboveground tree biomass of the site to be *c*. 28.5 kg m^−2^, which is in agreement with previous estimates conducted at Harvard Forest using multiple allometry methods (Ahmed *et al*., 2013). Combining this forest biomass estimate with measured total NSC concentrations from our five study species, we estimated average total NSC storage across the year at the ecosystem level to be 0.41 kg C m^−2^ or 1.03 kg NSC m^−2^. Of this total, 0.59 kg m^−2^ was sugars and 0.44 kg m^−2^ was starch. Partitioning total NSC storage across organs showed that the majority of NSCs were stored in the branches (0.40 kg m^−2^), followed by stemwood (0.27 kg m^−2^) and roots (0.32 kg m^−2^), with substantially less stored in foliage (0.04 kg m^−2^).

**Figure 6:**
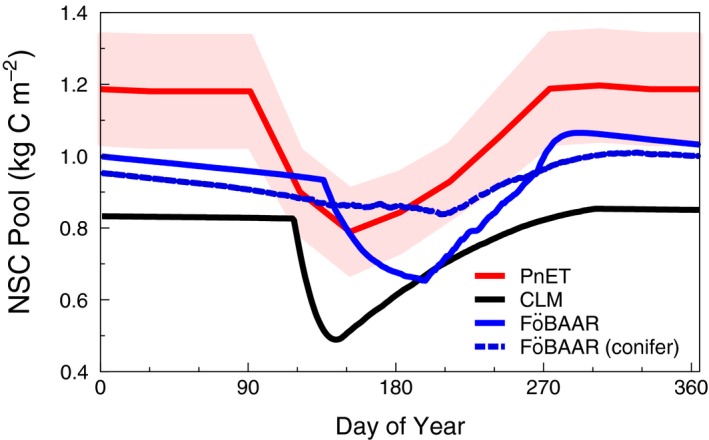
Seasonal variation in the total size of the ecosystem‐level nonstructural carbohydrate (NSC) storage pool (kg C m^−2^), using two ecosystem models (PnET, Aber *et al*., 1996; and FöBAAR, Keenan *et al*., 2012) and a land surface model (CLM, Levis *et al*., 2004). Model runs are assuming forest composition is 100% deciduous broadleaf tree species, except for dotted blue line which shows FöBAAR run assuming forest composition is 100% evergreen conifer species. Plotted values indicate means calculated across 15‐yr model run (1991–2004), using environmental data from Harvard Forest to drive the model. For PnET, uncertainty range illustrates interannual variation (± 1 SD) around the mean.

Additionally, estimated ecosystem‐level total NSC storage was in line with the allocation of assimilated C to various forest C pools (Table 1). Based on these estimates, stored NSCs at the ecosystem level could support woody biomass production for an entire year. Importantly, when comparing our results with predictions from ecosystem and land surface models, we find that all process‐based simulation models overpredict total NSC storage and seasonal depletion of NSC reserves at the ecosystem level for Harvard Forest (Fig. 6).

**Table 1:** Forest stand‐level characteristics at Harvard Forest

Component	Estimate	From	Reference
Total NSC	0.41 ± 0.05 kg C m^−2^	2014	Furze *et al*. (2018); Munger & Wofsy (2018)
Sugar	0.24 ± 0.05 kg C m^−2^	2014	Furze *et al*. (2018); Munger & Wofsy (2018)
Starch	0.18 ± 0.06 kg C m^−2^	2014	Furze *et al*. (2018); Munger & Wofsy (2018)
Total NSC relative to forest biomass	3.6 ± 0.4%	2014	Furze *et al*. (2018); Munger & Wofsy (2018)
Aboveground woody biomass increment	0.16 ± 0.03 kg C m^−2^ yr^−1^	1994–2014	Munger & Wofsy (2018)
Belowground woody biomass increment	0.04 kg C m^−2^ yr^−1^	2014	Furze *et al*. (2018); Munger & Wofsy (2018)
Total woody biomass increment	0.20 kg C m^−2^ yr^−1^	2014	Furze *et al*. (2018); Munger & Wofsy (2018)
Annual NEE of CO_2_	−0.24 ± 0.10 kg C m^−2^ yr^−1^	1992–2004	Urbanski *et al*. (2007)
Growing season NEE of CO_2_	−0.49 ± 0.11 kg C m^−2^ yr^−1^	1992–2004	Urbanski *et al*. (2007)
Dormant season NEE of CO_2_	0.24 ± 0.05 kg C m^−2^ yr^−1^	1992–2004	Urbanski *et al*. (2007)
Total NSC accumulation from April to October	0.14 kg C m^−2^	2014	Furze *et al*. (2018); Munger & Wofsy (2018)

NEE, net ecosystem exchange; NSC, nonstructural carbohydrates.

Uncertainty values are ± 1 SD of the mean.

## Discussion

### Size of whole‐tree pools, and differences among species

At the whole‐tree level, the amount of NSCs stored differed between species. In general, our results showed that whole‐tree total NSC storage fell along a gradient with larger pools for ring‐porous species compared with diffuse‐porous and evergreen. This finding is not surprising considering that deciduous species are thought to rely on stored reserves for springtime growth (Kramer & Kozlowski, 1979; Piispanen & Saranpää, 2001), and more specifically, ring‐porous deciduous species complete a large portion of new xylem formation using stored reserves before leaf expansion (Hinckley & Lassoie, 1981; Bréda & Granier, 1996). Thus, differences in the amount of NSC stored between species are expected based on these traits.

Throughout the year, whole‐tree sugar pools were often larger than starch pools. However, deciduous species had substantial starch reserves by October. Starch storage dominating the onset of the dormant season is in agreement with previous work that quantified whole‐tree pools for red oak (Richardson *et al*., 2015). Most studies, however, look at concentrations in individual organs rather than whole‐tree pools and have reported that starch concentrations were higher than sugar concentrations throughout the growing season, particularly in the branch sapwood (Hoch *et al*., 2003) and outer stem sapwood (Hoch *et al*., 2003; Richardson *et al*., 2013) of several temperate tree species. When examining the concentrations of individual organs, our results are in agreement with these studies. In the growing season, starch concentrations generally dominated the coarse roots and branches, and to a lesser extent, the outer 2 cm stemwood.

However, at the whole‐tree level, our results disagree and show support for sugar as the larger pool. This discrepancy is due to the inclusion of stemwood xylem to the pith in our whole‐tree pool estimates which increased sugars, but not starch. When taking the entire stemwood into consideration rather than just the outer sapwood like in previous studies, sugar concentrations generally dominated not only the growing season, but also the entire year. As stemwood is the largest biomass fraction, higher stemwood sugar concentrations contributed to larger overall sugar storage than starch storage at the whole‐tree level. Additionally, if we had ignored the radially varying concentrations of total NSCs and used those in the outer 2 cm stemwood and outer 1 cm root as proxies for whole‐stem and root storage, then mean annual whole‐tree total NSC storage would have been overestimated by 75% for our sample of 24 trees.

### Seasonal patterns of whole‐tree pools

Seasonal dynamics of whole‐tree total NSC pools were in agreement with the conventional theory of storage dynamics in which NSC reserves increase throughout the growing season and decrease throughout the dormant season. Interestingly, this seasonal pattern was not resolved when looking at the dynamics of individual organs, sugars, or starch alone; only when these components were combined did we find that whole‐tree storage dynamics aligned with the expected pattern despite seasonal interconversion between sugars and starch and within‐tree transport between organs.

In general, whole‐tree total NSC pools peaked in October for the deciduous species and in June for evergreen white pine, and then declined onward. The difference in timing of peak accumulation and depletion may be explained by growth characteristics. As white pine retains a majority of its needles throughout the dormant season, it has an advantage for C gain earlier in the growing season before new structural growth, leading to maximal NSC pools in June, and continued activity later into the autumn (Jurik *et al*., 1988). However, later bud break in evergreen conifers (O'Keefe, 2015), lower photosynthetic rates (Reich *et al*., 1995) and shoot apex development (Owston, 1969) along with xylem formation/thickening (Murmanis & Sachs, 1969) that both initiate later and continue well into the autumn may all contribute to the post‐June decline. Additionally, the decline may indicate excess C being allocated to root exudation (Abramoff & Finzi, 2016).

Even though NSCs cannot be translated into growth 1:1, for instance, due to metabolic losses, we found that the requirements for annual biomass production were in line with the magnitude of whole‐tree total NSC pool depletion. For the deciduous species, the annual production of foliage, branches, stemwood, and roots required 4.4–7.9 kg C, leading to an initial 1.8–3.0 kg C decline in the whole‐tree total NSC pool by April/June. By contrast, white pine's annual growth required 6.3 kg C, with an associated 2.2 kg C decline between June and October. It is often assumed that deciduous species will show a larger seasonal fluctuation in reserves than evergreen due to their reliance on stored NSCs for leaf out and xylem formation in the spring, (Kramer & Kozlowski, 1979; Piispanen & Saranpää, 2001). While our results suggest that evergreen species may be relying less on stored NSCs for new growth, seasonal fluctuations in whole‐tree total NSC pools were surprisingly similar. The mean minimum NSC storage in April was *c*. 75% of the maximum in October for each deciduous species and 70% of the maximum in June for evergreen white pine. Thus, larger pool size did not correlate with more pronounced seasonal fluctuation, and our results agree with previous reports that showed seasonal fluctuations were not always greater for deciduous species compared with evergreen (compiled in Martínez‐Vilalta *et al*., 2016).

### Storage dynamics at the organ level

We observed substantial differences in the amount of NSCs stored between belowground and aboveground organs. Branches were the largest reservoir of total NSCs and often sugars, and were also an important store of starch, accounting for *c*. 30–40% of total NSCs and 55–75% of starch stored throughout the whole‐tree at times of maximal NSC storage. Moreover, roots are thought to specialize as storage organs more so than any other organ (Loescher *et al*., 1990; Kozlowski, 1992), so we hypothesized that the largest starch pools would be found in the roots serving a longer term storage function. Interestingly, our results show that branches had a comparable starch pool throughout the year. However, organ‐level storage differed between species. For instance, red oak had a particularly large starch pool in the roots (mean annual 8 kg; effective mean concentration 44.0 mg g^−1^) compared with that of white pine (1 kg; 8.0 mg g^−1^). In general, deciduous species stored NSCs across organs, while white pine primarily stored its reserves aboveground.

Furthermore, the functional roles of sugars and starch were reflected in the seasonal patterns of each organ. Seasonal patterns of sugar pools were more similar between organs than starch pools. In general, sugar pools were higher in the dormant season than in the growing season for all organs, supporting an osmoprotective role for sugars throughout the tree in the wintertime (Ögren, 1997). Notably, while branch biomass represented *c*. 25% of aboveground biomass, sugar pools in the branches (mean 7.5 kg; effective mean concentration 53.0 mg g^−1^) were equal to or larger than those in stemwood (7.5 kg; 17.0 mg g^−1^) during cold months, suggesting that high sugar concentrations in the branches help to combat cold temperatures experienced in the canopy. By contrast, starch pools in aboveground organs generally increased over the growing season, providing evidence for starch's role in maintaining photosynthesis and phloem transport throughout the growing season (Paul & Foyer, 2001). Newly assimilated C is exported as sucrose and converted to starch for longer term storage to prevent inhibition of further transport, which would lead to the downregulation of photosynthesis.

As previously mentioned, roots were expected to have a high storage capacity, with reserves being used for annual growth (Loescher *et al*., 1990). Yet, total NSC pools in the roots of deciduous species remained fairly stable throughout the year, and accounted for 25–35% of whole‐tree total NSC reserves. In this way, roots may serve as a stable, longer term storage pool that is only drawn upon in catastrophic situations (i.e. loss of aboveground biomass), which would explain the older C previously reported in roots (Carbone *et al*., 2013; Richardson *et al*., 2015).

By contrast, early growing season declines were evident in the branches. Specifically, sugar pools in the branches declined between February and April, which is in accordance with branches serving as the closest source organ to support leaf out (Landhäusser & Lieffers, 2003). Our results suggest that declining branch reserves represent contributions to new leaf production rather than conversion of antifreeze sugar compounds back to starch because branch total NSC pools also declined indicating movement out of branches rather than conversion from sugars to starch within branches. For red oak, we estimated that new leaf production would require 7.6 kg C, and observed that total NSC pools in red oak branches declined by 70% (5.5 kg C) between February and April. While stored NSCs support both new foliage and wood production, elongation and thickening of foliage is to some degree supported by concurrent photosynthesis (Keel & Schädel, 2010).

As noted earlier, the seasonal fluctuation in total NSCs at the whole‐tree level was *c*. 30% for each species, suggesting minimal drawdown of stored reserves throughout the year. Martínez‐Vilalta *et al*. (2016) reported similar patterns and a suite of physiological and evolutionary reasons have been proposed to explain why trees maintain large reserves if they are rarely depleted by seasonal demands (Kobe, 1997; Millard *et al*., 2007; Adams *et al*., 2013; Carbone *et al*., 2013; O'Brien *et al*., 2014). However, while total NSC reserves may not be substantially drawn down across the seasons at the whole‐tree level, our results challenge this idea at the organ level. Notably, total NSCs in branches of red oak and white ash had substantial seasonal fluctuations. The total NSC pool in red oak branches declined by 70% during the winter and spring, and was replenished by autumn. A similar pattern was observed for white ash with a 50% decline.

### Estimation of ecosystem‐level total NSC storage

We estimated average total NSC storage across the year in a mixed temperate forest to be 1.03 kg NSCs m^−2^ or 0.41 kg C m^−2^, which falls within the 0.23–1.6 kg C m^−2^ range previously reported for total NSC storage at the whole‐forest level (reviewed in Dietze *et al*., 2014). This estimate corresponds to *c*. 4% of forest biomass compared to 8% revealed in the first ecosystem‐level estimate of total NSC storage in a semi‐deciduous tropical forest, which was 1.6 kg NSCs m^−2^ assuming a 20 kg m^−2^ woody biomass (Würth *et al*., 2005). The majority of total NSCs were stored aboveground in both forest types. However, a direct comparison is cautioned against due to differences between the two studies such as the ratio of sapwood to heartwood in the tree species studied as well as the biomass components used for upscaled estimates of total NSC storage. In our trees, contributions to total NSC storage differed between organs: branches (40%), stemwood (25%), roots (30%), and foliage (5%).

Few studies have modelled and validated NSC pool dynamics and C allocation to various sinks at the forest stand‐level, and when this validation has been done it has been with limited field measurements (Cropper & Gholz, 1993; Sampson *et al*., 2001; Gough *et al*., 2009). However, in this study, we combined high spatio‐temporal resolution field measurements of NSCs from multiple species over the course of a year with eddy flux tower and structural growth data to assess total NSC storage in the context of whole‐forest C balance at Harvard Forest. Over the course of the year, Harvard Forest exhibited net C uptake in the growing season and release in the dormant season. The mean annual rate of net ecosystem exchange (NEE) of CO_2_ was in substantial agreement with the annual total woody biomass increment, suggesting that most of the net C uptake was sequestered in long‐lived, woody biomass. However, during the growing season, there was greater net C assimilation than allocation to new woody biomass production, and ecosystem‐level total NSCs increased by 0.14 kg C m^−2^.

Estimates of total NSC pool size and seasonal dynamics were generated for Harvard Forest (Fig. 6; Richardson *et al*., 2012) using three process‐based simulation models (PnET, Aber *et al*., 1996; CLM, Levis *et al*., 2004; FöBAAR, Keenan *et al*., 2012). Model runs revealed 50% variation across models in estimates of total NSC pool size, a drawdown of the total NSC pool in spring and refilling over the summer, and similar pool size, but greatly reduced seasonal fluctuation for conifers relative to deciduous species. Notably, our results indicate that these process‐based models all overpredict the amount of stored NSCs, with twice as much being stored than was estimated based on extensive field measurements. While simulation models represent C assimilation, allocation, and metabolism, they do not consider controls on sink capacity and feedbacks between these processes, yielding a ‘source‐driven’ structure of C relations (Fatichi *et al*., 2014), and, ultimately, simpler dynamics than are expected for complex, living plants like trees.

Our allometric scaling accounted for varying concentrations between different organs and within individual organs, particularly the radial distribution of NSCs that occurs in the stemwood; this may explain the discrepancy between our scaled‐up estimates and process‐based model predictions at the ecosystem level. Typically, total NSC concentrations in the outer sapwood of the stem have been measured (Fajardo *et al*., 2013; Richardson *et al*., 2013). If we had scaled up based on concentrations in the outer 2 cm stemwood and 1 cm root, we would have estimated 0.72 kg C m^−2^ at the ecosystem level, which is closer to model‐based estimates, but 75% more than the value of 0.41 kg C m^−2^ which we obtained using radially varying NSC concentrations. If trees in the process‐based models had full access to a total NSC pool that was two times larger than was estimated based on field measurements, then trees might appear more resilient to disturbance and stress, and modelled productivity might be less sensitive to interannual variation in weather.

### Conclusions

We have constructed the most detailed assessment of NSC storage in temperate forest trees to date. We measured NSC concentrations in different organs, across multiple tree species, at a monthly time step over the course of year. We used allometric equations and forest inventory data to scale these concentrations up to the whole‐tree and whole‐ecosystem levels. Our results provide a picture of NSC storage in temperate forest trees in which: (1) The size of whole‐tree total NSC pools differed between species. (2) Whole‐tree total, sugar, and starch pools had contrasting seasonal patterns. (3) NSC storage and seasonal patterns differed between belowground and aboveground organs, with both (2) and (3) indicating different functional roles of sugars and starch and different contributions by individual organs to the overall whole‐tree C balance.

Notably, we showed that: (1) Deciduous ring‐porous species had larger whole‐tree total NSC pools than both deciduous diffuse‐porous and evergreen conifer species. (2)(a) The seasonal patterns of whole‐tree total NSC pools supported the conventional theory, but whole‐tree sugar pools were higher in the dormant season than the growing season, and whole‐tree starch pools peaked later in the growing season for deciduous species compared with evergreen white pine. (b) Although seasonal fluctuation in total NSCs was minimal at the whole‐tree level, with comparable seasonal depletion for deciduous and evergreen species, it was substantial at the organ level, particularly in the branches. (3) Roots were not the major storage organ for starch, as branches stored comparable amounts throughout the year, and root reserves were not depleted to support new springtime growth. Furthermore, we show that commonly used, process‐based ecosystem and land surface models all overpredict ecosystem‐level total NSC storage. Therefore, our results improve our understanding of C dynamics at both the whole‐tree and ecosystem levels and, importantly, resolve how the dynamics of individual organs contribute to the overall C balance.

## Author contributions

MEF, BAH, MSC and ADR planned the project. MEF and BAH conducted the field sampling with help from DMA, CDS and several generous lab members and collaborators. CDS contributed to sample preparation and methodology, MEF conducted NSC analyses and allometric scaling. MEF and ADR contributed to data analysis and interpretation of the results. MEF took the lead in writing the manuscript, with feedback and approval from co‐authors.

## Supporting information

Please note: Wiley Blackwell are not responsible for the content or functionality of any Supporting Information supplied by the authors. Any queries (other than missing material) should be directed to the *New Phytologist* Central Office.

**Fig. S1** Air temperature and precipitation data for Harvard Forest.
**Fig. S2** Comparison of estimated organ biomasses between five temperate tree species.
**Methods S1** NSC concentration measurements and uncertainty.
**Methods S2** Allometric scaling from NSC concentrations to whole‐tree pools.
**Methods S3** Estimation of foliar NSC pools.
**Methods S4** Estimation of ecosystem‐level NSC storage.
**Table S1** Diameter at breast height, height, and age for individual trees.
**Table S2** Estimated biomass of each organ for individual trees.
**Table S3** Tukey's HSD results from repeated measures linear mixed‐effects models testing for the effect of sampling month on whole‐tree total NSC, sugar, and starch pools for each species.
**Table S4** Results of repeated measures linear mixed‐effects models testing for the effect of organ, species, and their interaction on organ‐level total NSC, sugar, and starch pools.
**Table S5** Tukey's HSD results from repeated measures linear mixed‐effects models testing for the effect of organ on organ‐level NSC, sugar, and starch pools for each species.
**Table S6** Results of repeated measures linear mixed‐effects models testing for the effect of sampling month, species, and their interaction on total NSC, sugar, and starch pools in branch, stemwood, and root.
**Table S7** Tukey's HSD results from repeated measures linear mixed‐effects models testing for the effect of month on total NSC, sugar, and starch pools for each organ and species.
**Table S8** Partitioning of sugar and starch pools among woody organs and sampling months for each species.
