# Supplementary material for: Whole‐tree nonstructural carbohydrate storage and seasonal dynamics in five temperate species
Source: New Phytol. 2018 Oct 12;221(3):1466–77. doi: 10.1111/nph.15462 (PMC6587558; doi:10.1111/nph.15462)
Supplement: Supplementary file 1 — Fig. S1 Air temperature and precipitation data for Harvard Forest. Fig. S2 Comparison of estimated organ biomasses between five temperate tree species. Methods S1 NSC concentration measurements and uncertainty. Methods S2 Allometric scaling from NSC concentrations to whole‐tree pools. Methods S3 Estimation of foliar NSC pools. Methods S4 Estimation of ecosystem‐level NSC storage. Table S1 Diameter at breast height, height, and age for individual trees. Table S2 Estimated biomass of each organ for individual trees. Table S3 Tukey's HSD results from repeated measures linear mixed‐effects models testing for the effect of sampling month on whole‐tree total NSC, sugar, and starch pools for each species. Table S4 Results of repeated measures linear mixed‐effects models testing for the effect of organ, species, and their interaction on organ‐level total NSC, sugar, and starch pools. Table S5 Tukey's HSD results from repeated measures linear mixed‐effects models testing for the effect of organ on organ‐level NSC, sugar, and starch pools for each species. Table S6 Results of repeated measures linear mixed‐effects models testing for the effect of sampling month, species, and their interaction on total NSC, sugar, and starch pools in branch, stemwood, and root. Table S7 Tukey's HSD results from repeated measures linear mixed‐effects models testing for the effect of month on total NSC, sugar, and starch pools for each organ and species. Table S8 Partitioning of sugar and starch pools among woody organs and sampling months for each species. [file NPH-221-1466-s001.pdf]

***New Phytologist* Supporting Information**

Article title: Whole-tree nonstructural carbohydrate storage and seasonal dynamics in five temperate species

Authors: Morgan E. Furze, Brett A. Huggett, Donald M. Aubrecht, Claire D. Stolz, Mariah S. Carbone, and Andrew D. Richardson

Article acceptance date: 25 August 2018

The following Supporting Information is available for this article:

Figure S1 Air temperature and precipitation data for Harvard Forest

Figure S2 Comparison of estimated organ biomasses between five temperate tree species

Table S1 Diameter at breast height, height, and age for individual trees

Table S2 Estimated biomass of each organ for individual trees

Table S3 Tukey's HSD results from repeated measures linear mixed-effects models testing for the effect of sampling month on whole-tree total NSC, sugar, and starch pools for each species

Table S4 Results of repeated measures linear mixed-effects models testing for the effect of organ, species, and their interaction on organ-level A) total NSC, B) sugar, and C) starch pools

Table S5 Tukey's HSD results from repeated measures linear mixed-effects models testing for the effect of organ on organ-level NSC, sugar, and starch pools for each species

Table S6 Results of repeated measures linear mixed-effects models testing for the effect of sampling month, species, and their interaction on total NSC, sugar, and starch pools in A) branch, B) stemwood, and C) root

Table S7 Tukey's HSD results from repeated measures linear mixed-effects models testing for the effect of month on total NSC, sugar, and starch pools for each organ and species

Table S8 Partitioning of sugar and starch pools among woody organs and sampling months for each species

Methods S1 NSC concentration measurements and uncertainty

Methods S2 Allometric scaling from NSC concentrations to whole-tree pools

Methods S3 Estimation of foliar NSC pools

Methods S4 Estimation of ecosystem-level NSC storage

Figure S1 Average monthly A) air temperature and B) precipitation at Harvard Forest as measured by the Fisher Meteorological Station for the study year 2014 and the period 2002-2017. For a given year, average monthly air temperature was computed as the average of daily averages. Likewise, average monthly precipitation was computed as the average of the sum of daily totals. This included water equivalent of snow. Peach shading represents the average of each average monthly air temperature ± 1SD for the period 2002-2017. Blue shading represents the average of each average monthly precipitation ± 1SD for the period 2002-2017. Black dashed lines represent the monthly averages for our study year 2014. In C) daily minimum and maximum air temperatures are displayed. Shading represents the range between average daily minimum and maximum air temperature for the period 2002-2017 (dark gray), as well as daily minimum and maximum air temperature for the study year 2014 (orange-red). Light gray shading represents ± 1SD associated with average daily minimum/maximum air temperature for the period 2002-2017.

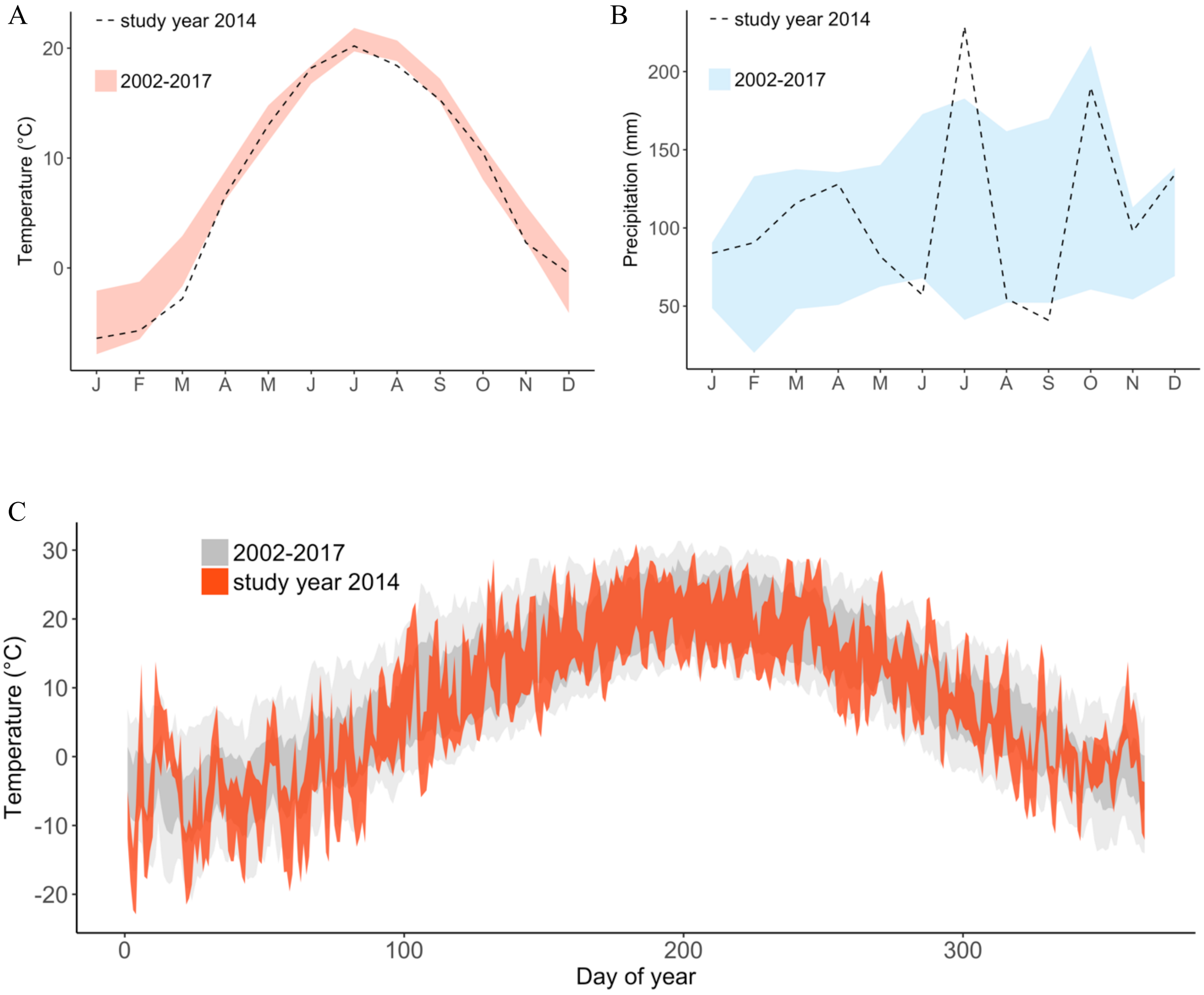

Figure S2 Comparison of estimated organ biomasses (branch, stemwood, and root) between red oak, white ash, red maple, paper birch, and white pine using allometric scaling theory. Error bars denote ± 1SE of the mean. Lowercase letters indicate significance of differences among species for branch biomass. Uppercase letters indicate significance of differences among organs. See Table S2 for estimated organ biomasses of individual trees.

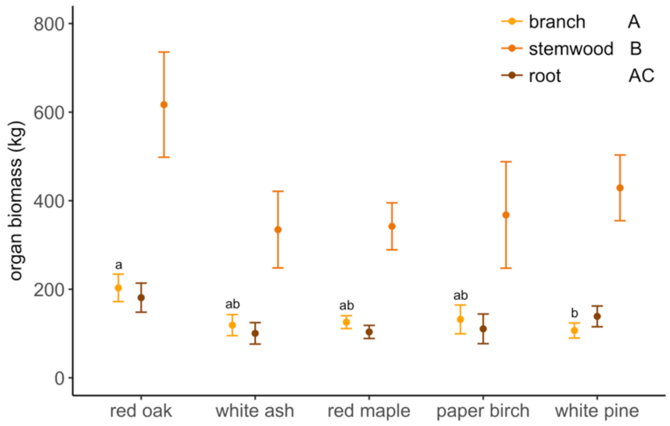

Note: We used a one-way ANOVA to individually analyze whole-tree biomass, branch biomass, stemwood biomass, and root biomass among species. Whole-tree biomass (not displayed) did not differ between species ($P = 0.17$). Organ biomass did not differ between species for roots ($P = 0.19$) and stemwood ($P = 0.18$), but it did for branches ($P = 0.048$, lowercase letters). We also used a one-way ANOVA to analyze biomass differences between organs ($P < 0.0001$, uppercase letters). For significant ANOVAs, differences between pairs of means were evaluated using Tukey's HSD, $\alpha = 0.05$, and results are displayed above with corresponding letters.

Table S1 Diameter at breast height, height, and age for individual trees; QURU=red oak, FRAM=white ash, ACRU=red maple, BEPA=paper birch, and PIST=white pine.

species	tree	DBH (cm)	height (m)	age (y)
FRAM	101	33.0	23.4	111
ACRU	102	39.5	22.0	61
ACRU	103	26.0	21.6	65
QURU	104	43.0	23.2	96
ACRU	105	29.0	20.7	86
BEPA	106	26.5	19.3	74
QURU	107	43.0	22.6	83
FRAM	108	39.5	19.5	91
QURU	109	46.0	18.6	108
BEPA	110	40.5	19.5	104
PIST	111	38.0	23.2	65
ACRU	112	33.0	21.0	72
BEPA	113	30.5	20.1	67
PIST	114	34.5	20.1	66
QURU	115	30.0	20.7	82

PIST	116	31.0	19.8	75
QURU	117	29.0	19.8	69
QURU	118	34.5	21.9	72
PIST	119	50.0	20.3	67
ACRU	120	34.0	21.7	97
PIST	121	41.0	20.9	80
PIST	122	44.5	21.3	87
FRAM	123	28.0	18.7	75
ACRU	124	30.0	19.8	68

Table S2 Estimated biomass (kg) of each organ using allometric scaling theory and standard coefficients and equations for individual trees. Total biomass is the sum of branch, stemwood, and coarse root; QURU=red oak, FRAM=white ash, ACRU=red maple, BEPA=paper birch, and PIST=white pine. See Methods S2 for details of allometric equations.

species	tree	root	stemwood	branch	total biomass
FRAM	101	93	309	113	515
ACRU	102	166	566	186	918
ACRU	103	62	196	84	343
QURU	104	238	821	257	1316
ACRU	105	81	259	103	443
BEPA	106	65	206	87	358
QURU	107	238	821	257	1316
FRAM	108	145	496	163	804
QURU	109	280	975	295	1550
BEPA	110	176	603	195	974
PIST	111	119	365	93	577
ACRU	112	109	360	132	601
BEPA	113	91	295	113	499
PIST	114	94	287	74	456
QURU	115	100	324	126	549
PIST	116	73	220	59	351
QURU	117	92	296	118	506
QURU	118	140	465	166	771
PIST	119	231	721	173	1124
ACRU	120	117	388	139	645
PIST	121	143	441	110	694
PIST	122	174	540	133	847
FRAM	123	62	199	81	343
ACRU	124	87	283	110	480

Table S3 Tukey's HSD results from repeated measures linear mixed-effects models testing for the effect of sampling month on whole-tree total NSC, sugar, and starch pools for each species. Individual tree biomass was included as a covariate. NS= not significant, * = $P \leq 0.05$, ** = $P \leq 0.01$, *** = $P \leq 0.001$, or **** = $P \leq 0.0001$ in each white cell indicates if column month and row month are significantly different from each other or not based on the difference between their least squares means. Each light gray cell contains this difference, calculated by row month minus column month. If the entire grid is shaded orange, then month was not a significant factor in the repeated measures linear mixed-effects model with $P \geq 0.05$ and post-hoc testing was not conducted.

RED OAK																				
whole-tree total NSC							whole-tree sugar							whole-tree starch						
	Jan	Apr	Jun	Aug	Oct	Dec		Jan	Apr	Jun	Aug	Oct	Dec		Jan	Apr	Jun	Aug	Oct	Dec
Jan							Jan		13.0	14.8	16.5	12.7	6.3	Jan		-6.3	-8.0	-14.9	-18.9	-5.6
Apr							Apr	****		1.8	3.5	-0.2	-6.7	Apr	NS		-1.7	-8.7	-12.7	0.6
Jun							Jun	****	NS		1.7	-2.0	-8.5	Jun	NS	NS		-7.0	-11	2.3
Aug							Aug	****	NS	NS		-3.7	-10.2	Aug	**	NS	NS		-4.0	9.3
Oct							Oct	****	NS	NS	NS		-6.5	Oct	***	*	NS	NS		13.3
Dec							Dec	NS	NS	**	**	NS		Dec	NS	NS	NS	NS	*	

WHITE ASH																				
whole-tree total NSC							whole-tree sugar							whole-tree starch						
	Jan	Apr	Jun	Aug	Oct	Dec		Jan	Apr	Jun	Aug	Oct	Dec		Jan	Apr	Jun	Aug	Oct	Dec
Jan							Jan		7.3	9.4	9.7	5.7	3.0	Jan						
Apr							Apr	*		2.1	2.4	-1.6	-4.4	Apr						
Jun							Jun	**	NS		0.3	-3.7	-6.5	Jun						
Aug							Aug	**	NS	NS		-4.0	-6.7	Aug						
Oct							Oct	NS	NS	NS	NS		-2.8	Oct						
Dec							Dec	NS	NS	NS	NS	NS		Dec						

RED MAPLE																				
whole-tree total NSC							whole-tree sugar							whole-tree starch						
	Jan	Apr	Jun	Aug	Oct	Dec		Jan	Apr	Jun	Aug	Oct	Dec		Jan	Apr	Jun	Aug	Oct	Dec
Jan		6.6	2.0	9.1	-1.4	4.1	Jan		7.9	9.3	11.1	8.3	3.3	Jan		-1.3	-7.3	-2.0	-9.6	0.8
Apr	*		-4.6	2.5	-8.0	-2.5	Apr	**		1.4	3.2	0.3	-4.6	Apr	NS		-6.0	-0.7	-8.3	2.1
Jun	NS	NS		7.1	-3.4	2.1	Jun	****	NS		1.7	-1.1	-6.0	Jun	***	**		5.3	-2.3	8.1
Aug	**	NS	*		-10.4	-4.9	Aug	****	NS	NS		-2.8	-7.8	Aug	NS	NS	*		-7.6	2.8
Oct	NS	**	NS	***		5.5	Oct	***	NS	NS	NS		-4.9	Oct	****	***	NS	***		10.3
Dec	NS	NS	NS	NS	NS		Dec	NS	NS	**	***	*		Dec	NS	NS	***	NS	****	

PAPER BIRCH																				
whole-tree total NSC							whole-tree sugar							whole-tree starch						
	Jan	Apr	Jun	Aug	Oct	Dec		Jan	Apr	Jun	Aug	Oct	Dec		Jan	Apr	Jun	Aug	Oct	Dec
Jan							Jan		5.1	8.5	7.0	5.6	3.5	Jan		-1.0	-4.3	-3.1	-8.5	0.4
Apr							Apr	NS		3.4	1.8	0.4	-1.6	Apr	NS		-3.3	-2.1	-7.5	1.5
Jun							Jun	*	NS		-1.5	-2.9	-5.0	Jun	NS	NS		1.2	-4.2	4.7
Aug							Aug	NS	NS	NS		-1.4	-3.4	Aug	NS	NS	NS		-5.3	3.6
Oct							Oct	NS	NS	NS	NS		-2.0	Oct	**	*	NS	NS		8.9
Dec							Dec	NS	NS	NS	NS	NS		Dec	NS	NS	NS	NS	**	

WHITE PINE																				
whole-tree total NSC							whole-tree sugar							whole-tree starch						
	Jan	Apr	Jun	Aug	Oct	Dec		Jan	Apr	Jun	Aug	Oct	Dec		Jan	Apr	Jun	Aug	Oct	Dec
Jan		1.1	-4.6	-0.5	1.0	-2.1	Jan		3.6	3.3	2.5	2.7	-0.5	Jan		-2.5	-8.0	-2.9	-1.7	-1.6
Apr	NS		-5.7	-1.5	-0.07	-3.1	Apr	**		-0.3	-1.1	-0.9	-4.1	Apr	*		-5.4	-0.4	0.8	0.9
Jun	**	***		4.2	5.6	2.6	Jun	*	NS		-0.9	-0.6	-3.8	Jun	****	****		5.0	6.3	6.4
Aug	NS	NS	*		1.5	-1.6	Aug	NS	NS	NS		0.	-2.9	Aug	**	NS	****		1.2	1.3
Oct	NS	NS	***	NS		-3.1	Oct	NS	NS	NS	NS		-3.2	Oct	NS	NS	****	NS		0.1
Dec	NS	NS	NS	NS	NS		Dec	NS	**	**	*	*		Dec	NS	NS	****	NS	NS	

Table S4 Results of repeated measures linear mixed-effects models testing for the effect of organ, species, and their interaction on organ-level A) total NSC, B) sugar, and C) starch pools for 24 trees sampled at Harvard Forest. Individual organ biomass was included as a covariate.

	organ-level		
	A) total NSC	B) sugar	C) starch
organ	**<0.0001**	**<0.0001**	**<0.0001**
species	**<0.0001**	**0.0001**	**<0.0001**
o x s	**<0.0001**	**<0.0001**	**0.0001**

Table S5 Tukey's HSD results from repeated measures linear mixed-effects models testing for the effect of organ (branch, stemwood, root) on organ-level total NSC, sugar, and starch pools for each species. Individual organ biomass was included as a covariate. NS= not significant, * = $P \leq 0.05$, ** = $P \leq 0.01$, *** = $P \leq 0.001$, or **** = $P \leq 0.0001$ in each white cell indicates if column organ and row organ are significantly different from each other or not based on the difference between their least squares means. Each light gray cell contains this difference, calculated by row month minus column month. If the entire grid is shaded orange, then month was not a significant factor in the repeated measures linear mixed-effects model with $P \geq 0.05$ and post-hoc testing was not conducted.

RED OAK											
total NSC				sugar				starch			
	branch	stemwood	root		branch	stemwood	root		branch	stemwood	root
branch		10.2	0.7	branch		4.4	2.1	branch		5.8	-1.3
stemwood	****		-9.5	stemwood	***		-2.3	stemwood	**		-7.1
root	NS	****		root	*	NS		root	NS	***	
WHITE ASH											
total NSC				sugar				starch			
	branch	stemwood	root		branch	stemwood	root		branch	stemwood	root
branch		4.9	4.3	branch		3.7	4.0	branch		4.0	0.05
stemwood	NS		-0.6	stemwood	*		0.3	stemwood	*		-3.9
root	***	NS		root	****	NS		root	NS	*	
RED MAPLE											
total NSC				sugar				starch			
	branch	stemwood	root		branch	stemwood	root		branch	stemwood	root
branch		6.3	-0.2	branch		3.2	0.6	branch		4.9	-1.0
stemwood	***		-6.5	stemwood	***		-2.5	stemwood	****		-6.0
root	NS	***		root	NS	*		root	NS	****	
PAPER BIRCH											
total NSC				sugar				starch			
	branch	stemwood	root		branch	stemwood	root		branch	stemwood	root
branch		8.2	4.1	branch		4.2	3.8	branch			
stemwood	****		-4.1	stemwood	***		-0.3	stemwood			
root	***	*		root	****	NS		root			
WHITE PINE											
total NSC				sugar				starch			
	branch	stemwood	root		branch	stemwood	root		branch	stemwood	root
branch		4.0	5.9	branch		3.4	5.0	branch		1.7	1.0
stemwood	****		1.9	stemwood	****		1.6	stemwood	**		-0.7
root	****	*		root	****	*		root	**	NS	

Table S6 Results of repeated measures linear mixed-effects models testing for the effect of sampling month, species, and their interaction on total NSC, sugar, and starch pools in A) branch, B) stemwood, and C) root for 24 trees sampled at Harvard Forest. Individual organ biomass was included as a covariate.

	A) branch			B) stemwood			C) root		
	total NSC	sugar	starch	total NSC	sugar	starch	total NSC	sugar	starch
month	**<0.0001**	**<0.0001**	**<0.0001**	**<0.0001**	**<0.0001**	**<0.0001**	**<0.001**	**<0.0001**	**0.0001**
species	**<0.01**	**0.0001**	**<0.01**	**0.0001**	**<0.0001**	**<0.01**	**<0.0001**	**<0.0001**	**0.0001**
m x s	**<0.001**	**0.02**	**<0.0001**	**<0.0001**	**<0.01**	**<0.001**	0.91	**0.0001**	0.99

Table S7 Tukey's HSD results from repeated measures linear mixed-effects models testing for the effect of month on total NSC, sugar, and starch pools for each organ and species. Individual organ biomass was included as a covariate. 6-month data were used for roots whereas 12-month data were used for branches and stemwood. NS= not significant, * = $P \leq 0.05$, ** = $P \leq 0.01$, *** = $P \leq 0.001$, or **** = $P \leq 0.0001$ in each white cell indicates if column month and row month are significantly different from each other or not based on the difference between their least squares means. Each light gray cell contains this difference, calculated by row month minus column month. If the entire grid is shaded orange, then month was not a significant factor in the repeated measures linear mixed-effects model with $P \geq 0.05$ and post-hoc testing was not conducted.

RED OAK - ROOT																				
total NSC							sugar							starch						
	Jan	Apr	Jun	Aug	Oct	Dec		Jan	Apr	Jun	Aug	Oct	Dec		Jan	Apr	Jun	Aug	Oct	Dec
Jan							Jan		3.4	5.6	5.3	3.8	4.9	Jan						
Apr							Apr	*		2.2	1.9	0.5	1.5	Apr						
Jun							Jun	***	NS		-0.3	-1.7	-0.6	Jun						
Aug							Aug	***	NS	NS		-1.4	-0.4	Aug						
Oct							Oct	*	NS	NS	NS		1.1	Oct						
Dec							Dec	***	NS	NS	NS	NS		Dec						
WHITE ASH - ROOT																				
total NSC							sugar							starch						
	Jan	Apr	Jun	Aug	Oct	Dec		Jan	Apr	Jun	Aug	Oct	Dec		Jan	Apr	Jun	Aug	Oct	Dec
Jan							Jan		0.8	1.1	1.1	0.2	-0.7	Jan						
Apr							Apr	NS		0.3	0.3	-0.6	-1.5	Apr						
Jun							Jun	NS	NS		-0.02	-0.9	-1.8	Jun						
Aug							Aug	NS	NS	NS		-0.9	-1.8	Aug						
Oct							Oct	NS	NS	NS	NS		-0.9	Oct						
Dec							Dec	NS	NS	*	*	NS		Dec						
RED MAPLE - ROOT																				
total NSC							sugar							starch						
	Jan	Apr	Jun	Aug	Oct	Dec		Jan	Apr	Jun	Aug	Oct	Dec		Jan	Apr	Jun	Aug	Oct	Dec
Jan		1.1	-1.4	3.3	1.6	4.1	Jan		2.0	1.3	2.3	1.6	1.0	Jan		-0.9	-2.7	1.1	-0.01	3.1
Apr	NS		-2.5	2.4	0.5	3.0	Apr	*		-0.7	0.3	-0.4	-1.0	Apr	NS		-1.8	2.0	0.9	4.0
Jun	NS	NS		4.8	3.0	5.5	Jun	NS	NS		1.0	0.3	-0.3	Jun	NS	NS		3.8	2.7	5.8
Aug	*	NS	**		-1.7	0.7	Aug	**	NS	NS		-0.7	-1.2	Aug	NS	NS	**		-1.1	1.9
Oct	NS	NS	NS	NS		2.5	Oct	NS	NS	NS	NS		-0.6	Oct	NS	NS	NS	NS		3.1
Dec	*	NS	***	NS	NS		Dec	NS	NS	NS	NS	NS		Dec	*	**	****	NS	*	

PAPER BIRCH – ROOT																				
total NSC							sugar							starch						
	Jan	Apr	Jun	Aug	Oct	Dec		Jan	Apr	Jun	Aug	Oct	Dec		Jan	Apr	Jun	Aug	Oct	Dec
Jan							Jan							Jan						
Apr							Apr							Apr						
Jun							Jun							Jun						
Aug							Aug							Aug						
Oct							Oct							Oct						
Dec							Dec							Dec						
WHITE PINE - ROOT																				
total NSC							sugar							starch						
	Jan	Apr	Jun	Aug	Oct	Dec		Jan	Apr	Jun	Aug	Oct	Dec		Jan	Apr	Jun	Aug	Oct	Dec
Jan		0.3	-1.2	1.5	1.1	0.6	Jan							Jan		0.05	-1.3	0.7	0.7	0.6
Apr	NS		-1.5	1.2	0.8	0.3	Apr							Apr	NS		-1.3	0.7	0.7	0.6
Jun	NS	*		2.7	2.4	1.8	Jun							Jun	****	****		2.0	2.0	1.9
Aug	NS	NS	****		-0.4	-0.9	Aug							Aug	*	NS	****		-0.02	-0.1
Oct	NS	NS	***	NS		-0.5	Oct							Oct	NS	NS	****	NS		-0.08
Dec	NS	NS	*	NS	NS		Dec							Dec	NS	NS	****	NS	NS	

Table S7 continued on next page with vertical table orientation for stemwood and branches due to pairwise comparisons for 12-month data rather than 6-month data

RED OAK - STEMWOOD												
total NSC												
	Jan	Feb	Mar	Apr	May	Jun	Jul	Aug	Sept	Oct	Nov	Dec
Jan		-1.3	0.9	-1.2	3.2	0.7	0.8	2.4	-2.0	-0.5	-1.6	-4.1
Feb	NS		2.2	0.07	4.5	2.0	2.1	3.7	-0.7	0.8	-0.3	-2.8
Mar	NS	NS		-2.1	2.3	-0.2	-0.06	1.5	-2.9	-1.4	-2.5	-5.0
Apr	NS	NS	NS		4.4	1.9	2.0	3.6	-0.8	0.7	-0.4	-2.9
May	*	***	NS	***		-2.5	-2.3	-0.8	-5.1	-3.7	-4.8	-7.3
Jun	NS	NS	NS	NS	NS		0.1	1.7	-2.7	-1.2	-2.3	-4.8
Jul	NS	NS	NS	NS	NS	NS		1.6	-2.8	-1.4	-2.4	-4.9
Aug	NS	**	NS	**	NS	NS	NS		-4.4	-2.9	-4.0	-6.5
Sept	NS	NS	NS	NS	****	NS	NS	***		1.5	0.4	-2.1
Oct	NS	NS	NS	NS	**	NS	NS	NS	NS		-1.1	-3.6
Nov	NS	NS	NS	NS	****	NS	NS	**	NS	NS		-2.5
Dec	**	NS	****	NS	****	****	****	****	NS	**	NS	

RED OAK - STEMWOOD												
sugar												
	Jan	Feb	Mar	Apr	May	Jun	Jul	Aug	Sept	Oct	Nov	Dec
Jan		-2.1	0.2	1.7	2.3	3.3	2.5	3.1	1.6	2.1	0.009	-1.0
Feb	*		2.2	3.7	4.4	5.4	4.5	5.2	3.7	4.2	2.1	1.1
Mar	NS	**		1.5	2.1	3.2	2.3	2.9	1.4	1.9	-0.1	-1.1
Apr	NS	****	NS		0.6	1.7	0.8	1.4	-0.06	0.4	-1.6	-2.6
May	**	****	*	NS		1.0	0.2	0.8	-0.7	-0.2	-2.3	-3.3
Jun	****	****	****	NS	NS		-0.8	-0.2	-1.7	-1.2	-3.3	-4.3
Jul	**	****	**	NS	NS	NS		0.6	-0.9	-0.4	-2.5	-3.4
Aug	****	****	****	NS	NS	NS	NS		-1.5	-1.0	-3.1	-4.1
Sept	NS	****	NS	NS	NS	NS	NS	NS		0.5	-1.6	-2.6
Oct	*	****	*	NS	NS	NS	NS	NS	NS		-2.1	-3.1
Nov	NS	*	NS	NS	**	****	**	****	NS	*		-1.0
Dec	NS	NS	NS	***	****	****	****	****	**	****	NS	

RED OAK - STEMWOOD												
starch												
	Jan	Feb	Mar	Apr	May	Jun	Jul	Aug	Sept	Oct	Nov	Dec
Jan		0.8	0.7	-2.8	0.9	-2.6	-1.7	-0.7	-3.6	-2.6	-1.6	-3.1
Feb	NS		-0.05	-3.7	0.09	-3.4	-2.4	-1.5	-4.4	-3.4	-2.4	-3.9
Mar	NS	NS		-3.6	0.1	-3.3	-2.4	-1.4	-4.3	-3.3	-2.4	-3.8
Apr	**	****	****		3.8	0.3	1.2	2.2	-0.7	0.3	1.2	-0.2
May	NS	NS	NS	****		-3.5	-2.5	-1.6	-4.5	-3.5	-2.5	-4.0
Jun	*	***	***	NS	****		0.96	1.9	-1.0	0.01	1.0	-0.5
Jul	NS	*	*	NS	*	NS		0.9	-1.9	-1.0	0.02	-1.5
Aug	NS	NS	NS	NS	NS	NS	NS		-2.9	-1.9	-0.9	-2.4
Sept	***	****	****	NS	****	NS	NS	**		1.0	2.0	0.5
Oct	*	***	***	NS	****	NS	NS	NS	NS		1.0	-0.5
Nov	NS	*	*	NS	*	NS	NS	NS	NS	NS		-1.5
Dec	***	****	****	NS	****	NS	NS	*	NS	NS	NS	

WHITE ASH - STEMWOOD

total NSC

	Jan	Feb	Mar	Apr	May	Jun	Jul	Aug	Sept	Oct	Nov	Dec
Jan		1.1	1.7	0.8	4.0	1.4	3.2	3.1	1.7	0.8	-2.5	0.7
Feb	NS		0.6	-0.3	2.9	0.3	2.1	2.0	0.6	-0.3	-3.6	-0.4
Mar	NS	NS		-0.9	2.3	-0.3	1.5	1.4	-0.02	-0.9	-4.2	-1.0
Apr	NS	NS	NS		3.2	0.6	2.3	2.3	0.8	-0.06	-3.4	-0.2
May	*	NS	NS	NS		-2.6	-0.8	-0.9	-2.3	-3.2	-6.5	-3.3
Jun	NS	NS	NS	NS	NS		1.8	1.7	0.3	-0.6	-3.9	-0.7
Jul	NS	NS	NS	NS	NS	NS		-0.04	-1.5	-2.4	-5.7	-2.5
Aug	NS	NS	NS	NS	NS	NS	NS		-1.5	-2.4	-5.7	-2.5
Sept	NS	NS	NS	NS	NS	NS	NS	NS		-0.9	-4.2	-1.0
Oct	NS	NS	NS	NS	NS	NS	NS	NS	NS		-3.3	-0.1
Nov	NS	NS	*	NS	****	*	***	***	*	NS		3.2
Dec	NS	NS	NS	NS	NS	NS	NS	NS	NS	NS	NS	

WHITE ASH - STEMWOOD

sugar

	Jan	Feb	Mar	Apr	May	Jun	Jul	Aug	Sept	Oct	Nov	Dec
Jan		-0.6	-0.02	1.5	2.3	2.7	1.8	2.7	2.1	1.2	1.2	0.8
Feb	NS		0.6	2.1	2.9	3.3	2.4	3.3	2.7	1.8	1.8	1.4
Mar	NS	NS		1.5	2.3	2.7	1.8	2.7	2.1	1.2	1.2	0.8
Apr	NS	NS	NS		0.8	1.2	0.3	1.2	0.6	-0.3	-0.3	-0.7
May	NS	*	NS	NS		0.4	-0.5	0.4	-0.2	-1.1	-1.1	-1.5
Jun	NS	*	NS	NS	NS		-0.9	0.01	-0.6	-1.5	-1.5	-1.9
Jul	NS	NS	NS	NS	NS	NS		0.9	0.3	-0.6	-0.6	-1.0
Aug	NS	*	NS	NS	NS	NS	NS		-0.6	-1.5	-1.5	-1.9
Sept	NS	NS	NS	NS	NS	NS	NS	NS		-0.9	-0.9	-1.3
Oct	NS	NS	NS	NS	NS	NS	NS	NS	NS		-0.02	-0.4
Nov	NS	NS	NS	NS	NS	NS	NS	NS	NS	NS		-0.4
Dec	NS	NS	NS	NS	NS	NS	NS	NS	NS	NS	NS	

WHITE ASH - STEMWOOD

starch

	Jan	Feb	Mar	Apr	May	Jun	Jul	Aug	Sept	Oct	Nov	Dec
Jan		1.7	1.7	-0.7	1.7	-1.3	1.4	0.4	-0.4	-0.4	-3.7	-0.1
Feb	NS		$1.3e^{-15}$	-2.4	$-2.2e^{-16}$	-3.0	-0.3	-1.3	-2.1	-2.1	-5.4	-1.8
Mar	NS	NS		-2.4	$-1.6e^{-15}$	-3.0	-0.3	-1.3	-2.1	-2.1	-5.4	-1.8
Apr	NS	NS	NS		2.4	-0.6	2.1	1.1	0.3	0.3	-3.0	0.6
May	NS	NS	NS	NS		-3.0	-0.3	-1.3	-2.1	-2.1	-5.4	-1.8
Jun	NS	**	**	NS	**		2.7	1.7	0.9	0.8	-2.4	1.2
Jul	NS	NS	NS	NS	NS	*		-0.9	-1.8	-1.8	-5.1	-1.5
Aug	NS	NS	NS	NS	NS	NS	NS		-0.9	-0.9	-4.1	-0.6
Sept	NS	NS	NS	NS	NS	NS	NS	NS		-0.01	-3.3	0.3
Oct	NS	NS	NS	NS	NS	NS	NS	NS	NS		-3.3	0.3
Nov	***	****	****	**	****	****	****	***	**	**		3.6
Dec	NS	NS	NS	NS	NS	NS	NS	NS	NS	NS	**	

RED MAPLE - STEMWOOD												
total NSC												
	Jan	Feb	Mar	Apr	May	Jun	Jul	Aug	Sept	Oct	Nov	Dec
Jan		2.5	2.7	2.2	5.7	3.9	5.4	5.3	3.9	3.4	0.06	0.03
Feb	NS		0.3	-0.3	3.2	1.4	2.9	2.8	1.4	0.9	-2.4	-2.4
Mar	NS	NS		-0.5	2.9	1.2	2.7	2.6	1.2	0.7	-2.6	-2.7
Apr	NS	NS	NS		3.5	1.7	3.2	3.1	1.7	1.2	-2.1	-2.2
May	****	NS	NS	*		-1.8	-0.3	-0.4	-1.8	-2.3	-5.6	-5.6
Jun	**	NS	NS	NS	NS		1.5	1.4	0.01	-0.5	-3.8	-3.8
Jul	****	NS	NS	NS	NS	NS		-0.07	-1.5	-2.0	-5.3	-5.3
Aug	****	NS	NS	NS	NS	NS	NS		-1.4	-1.9	-5.2	-5.3
Sept	**	NS	NS	NS	NS	NS	NS	NS		-0.5	-3.8	-3.8
Oct	*	NS	NS	NS	NS	NS	NS	NS	NS		-3.3	-3.4
Nov	NS	NS	NS	NS	****	NS	****	****	**	*		-0.03
Dec	NS	NS	NS	NS	****	NS	****	****	**	*	NS	

RED MAPLE - STEMWOOD												
sugar												
	Jan	Feb	Mar	Apr	May	Jun	Jul	Aug	Sept	Oct	Nov	Dec
Jan		0.5	1.3	2.8	3.0	4.1	3.4	4.2	3.6	2.8	2.6	0.8
Feb	NS		0.8	2.3	2.5	3.6	2.9	3.7	3.1	2.4	2.1	0.3
Mar	NS	NS		1.5	1.7	2.9	2.2	3.0	2.4	1.6	1.4	-0.4
Apr	****	**	NS		0.2	1.4	0.7	1.5	0.9	0.1	-0.1	-1.9
May	****	***	*	NS		1.2	0.4	1.3	0.6	-0.1	-0.3	-2.1
Jun	****	****	****	NS	NS		-0.7	0.1	-0.5	-1.2	-1.5	-3.3
Jul	****	****	**	NS	NS	NS		0.8	0.2	-0.6	-0.8	-2.6
Aug	****	****	****	NS	NS	NS	NS		-0.6	-1.4	-1.6	-3.4
Sept	****	****	**	NS	NS	NS	NS	NS		-0.8	-1.0	-2.8
Oct	****	**	NS	NS	NS	NS	NS	NS	NS		-0.2	-2.0
Nov	***	**	NS	NS	NS	NS	NS	NS	NS	NS		-1.8
Dec	NS	NS	NS	*	**	****	***	****	****	*	*	

RED MAPLE - STEMWOOD												
starch												
	Jan	Feb	Mar	Apr	May	Jun	Jul	Aug	Sept	Oct	Nov	Dec
Jan		2.0	1.5	-0.6	2.7	-0.3	2.0	1.1	0.2	0.5	-2.6	-0.8
Feb	NS		-0.5	-2.5	0.7	-2.2	-0.01	-0.9	-1.7	-1.4	-4.5	-2.8
Mar	NS	NS		-2.0	1.2	-1.7	0.5	-0.4	-1.2	-0.9	-4.0	-2.3
Apr	NS	NS	NS		3.2	0.3	2.5	1.6	0.8	1.1	-2.0	-0.2
May	*	NS	NS	**		-2.9	-0.7	-1.6	-2.4	-2.1	-5.2	-3.5
Jun	NS	NS	NS	NS	*		2.2	1.3	0.5	0.8	-2.3	-0.5
Jul	NS	NS	NS	NS	NS	NS		-0.9	-1.7	-1.4	-4.5	-2.8
Aug	NS	NS	NS	NS	NS	NS	NS		-0.8	-0.5	-3.6	-1.9
Sept	NS	NS	NS	NS	NS	NS	NS	NS		0.3	-2.9	-1.1
Oct	NS	NS	NS	NS	NS	NS	NS	NS	NS		-3.1	-1.4
Nov	NS	****	****	NS	****	NS	****	***	*	**		1.8
Dec	NS	*	NS	NS	**	NS	*	NS	NS	NS	NS	

PAPER BIRCH - STEMWOOD												
total NSC												
	Jan	Feb	Mar	Apr	May	Jun	Jul	Aug	Sept	Oct	Nov	Dec
Jan		1.2	1.1	1.6	3.5	2.9	3.5	3.2	3.3	2.1	-2.3	0.7
Feb	NS		-0.1	0.4	2.3	1.7	2.3	2.0	2.1	0.9	-3.5	-0.5
Mar	NS	NS		0.5	2.4	1.9	2.5	2.2	2.2	1.0	-3.3	-0.4
Apr	NS	NS	NS		1.9	1.4	2.0	1.7	1.8	0.5	-3.8	-0.9
May	**	NS	NS	NS		-0.6	0.06	-0.2	-0.2	-1.4	-5.7	-2.8
Jun	*	NS	NS	NS	NS		0.6	0.3	0.4	-0.9	-5.2	-2.2
Jul	**	NS	NS	NS	NS	NS		-0.3	-0.2	-1.5	-5.8	-2.8
Aug	**	NS	NS	NS	NS	NS	NS		0.08	-1.2	-5.5	-2.5
Sept	**	NS	NS	NS	NS	NS	NS	NS		-1.2	-5.6	-2.6
Oct	NS	NS	NS	NS	NS	NS	NS	NS	NS		-4.3	-1.4
Nov	NS	**	**	***	****	****	****	****	****	****		3.0
Dec	NS	NS	NS	NS	*	NS	*	*	*	NS	**	

PAPER BIRCH - STEMWOOD												
sugar												
	Jan	Feb	Mar	Apr	May	Jun	Jul	Aug	Sept	Oct	Nov	Dec
Jan		0.01	0.2	2.0	2.3	3.1	2.4	2.9	2.7	2.3	2.8	1.4
Feb	NS		0.2	2.0	2.3	3.1	2.3	2.9	2.7	2.3	2.8	1.4
Mar	NS	NS		1.8	2.1	2.9	2.1	2.6	2.5	2.1	2.6	1.2
Apr	NS	NS	NS		0.3	1.1	0.4	0.9	0.7	0.3	0.9	-0.6
May	NS	NS	NS	NS		0.8	0.06	0.6	0.4	0.01	0.5	-0.9
Jun	**	**	**	NS	NS		-0.8	-0.2	-0.4	-0.8	-0.3	-1.7
Jul	NS	NS	NS	NS	NS	NS		0.5	0.3	-0.05	0.5	-1.0
Aug	**	**	*	NS	NS	NS	NS		-0.2	-0.6	-0.04	-1.5
Sept	*	*	*	NS	NS	NS	NS	NS		-0.4	0.1	-1.3
Oct	NS	NS	NS	NS	NS	NS	NS	NS	NS		0.5	-0.9
Nov	*	*	*	NS	NS	NS	NS	NS	NS	NS		-1.4
Dec	NS	NS	NS	NS	NS	NS	NS	NS	NS	NS	NS	

PAPER BIRCH - STEMWOOD												
starch												
	Jan	Feb	Mar	Apr	May	Jun	Jul	Aug	Sept	Oct	Nov	Dec
Jan		1.2	0.8	-0.4	1.2	-0.2	1.2	0.4	0.6	-0.2	-5.1	-0.7
Feb	NS		-0.3	-1.6	$1.9e^{-15}$	-1.4	$2.4e^{-15}$	-0.8	-0.6	-1.4	-6.3	-1.9
Mar	NS	NS		-1.3	-0.3	-1.0	-0.3	-0.5	-0.2	-1.1	-6.0	-1.6
Apr	NS	NS	NS		1.6	0.3	1.6	0.8	1.0	0.2	-4.7	-0.3
May	NS	NS	NS	NS		-1.4	$4.4e^{-16}$	-0.8	-0.6	-1.4	-6.3	-1.9
Jun	NS	NS	NS	NS	NS		1.4	0.5	0.8	-0.04	-4.9	-0.5
Jul	NS	NS	NS	NS	NS	NS		-0.8	-0.6	-1.4	-6.3	-1.9
Aug	NS	NS	NS	NS	NS	NS	NS		0.3	-0.6	-5.5	-1.1
Sept	NS	NS	NS	NS	NS	NS	NS	NS		-0.8	-5.7	-1.3
Oct	NS	NS	NS	NS	NS	NS	NS	NS	NS		-4.9	-0.5
Nov	****	****	****	****	****	****	****	****	****	****		4.4
Dec	NS	NS	NS	NS	NS	NS	NS	NS	NS	NS	****	

WHITE PINE - STEMWOOD												
total NSC												
	Jan	Feb	Mar	Apr	May	Jun	Jul	Aug	Sept	Oct	Nov	Dec
Jan		-0.1	0.2	-0.2	1.0	-0.8	0.4	0.3	-0.002	-0.1	-0.6	-2.9
Feb	NS		0.4	-0.07	1.1	-0.6	0.6	0.4	0.1	0.03	-0.5	-2.7
Mar	NS	NS		-0.4	0.7	-1.0	0.2	0.05	-0.2	-0.3	-0.8	-3.1
Apr	NS	NS	NS		1.2	-0.6	0.6	0.5	0.2	0.1	-0.4	-2.6
May	NS	NS	NS	NS		-1.8	-0.5	-0.7	-1.0	-1.1	-1.6	-3.8
Jun	NS	NS	NS	NS	*		1.2	1.0	0.8	0.7	0.2	-2.1
Jul	NS	NS	NS	NS	NS	NS		-0.2	-0.4	-0.5	-1.0	-3.3
Aug	NS	NS	NS	NS	NS	NS	NS		-0.3	-0.4	-0.9	-3.1
Sept	NS	NS	NS	NS	NS	NS	NS	NS		-0.1	-0.6	-2.8
Oct	NS	NS	NS	NS	NS	NS	NS	NS	NS		-0.5	-2.7
Nov	NS	NS	NS	NS	*	NS	NS	NS	NS	NS		-2.2
Dec	****	****	****	****	****	**	****	****	****	****	***	

WHITE PINE - STEMWOOD												
sugar												
	Jan	Feb	Mar	Apr	May	Jun	Jul	Aug	Sept	Oct	Nov	Dec
Jan		-0.2	0.2	1.0	1.0	0.9	0.6	1.0	0.4	0.6	0.3	-1.0
Feb	NS		0.4	1.2	1.1	1.1	0.8	1.1	0.6	0.8	0.5	-0.8
Mar	NS	NS		0.8	0.8	0.7	0.4	0.8	0.2	0.4	0.1	-1.2
Apr	**	***	*		-0.05	-0.1	-0.4	-0.1	-0.6	-0.4	-0.7	-2.0
May	**	***	NS	NS		-0.05	-0.3	-0.01	-0.6	-0.3	-0.7	-1.9
Jun	**	***	NS	NS	NS		-0.3	0.04	-0.5	-0.3	-0.6	-1.9
Jul	NS	*	NS	NS	NS	NS		0.3	-0.2	0.02	-0.3	-1.6
Aug	**	***	NS	NS	NS	NS	NS		-0.6	-0.3	-0.7	-1.9
Sept	NS	NS	NS	NS	NS	NS	NS	NS		0.2	-0.1	-1.4
Oct	NS	*	NS	NS	NS	NS	NS	NS	NS		-0.4	-1.6
Nov	NS	NS	NS	NS	NS	NS	NS	NS	NS	NS		-1.3
Dec	**	*	***	****	****	****	****	****	****	****	****	

WHITE PINE - STEMWOOD												
starch												
	Jan	Feb	Mar	Apr	May	Jun	Jul	Aug	Sept	Oct	Nov	Dec
Jan		0.03	0.02	-1.2	-0.1	-1.7	-0.2	-0.7	-0.4	-0.7	-0.9	-1.9
Feb	NS		-0.01	-1.3	-0.04	-1.7	-0.2	-0.8	-0.4	-0.8	-0.9	-1.9
Mar	NS	NS		-1.3	-0.03	-1.7	-0.2	-0.7	-0.4	-0.8	-0.9	-1.9
Apr	NS	NS	NS		1.2	-0.5	1.0	0.5	0.8	0.5	0.3	-0.6
May	NS	NS	NS	NS		-1.7	-0.2	-0.7	-0.4	-0.7	-0.9	-1.9
Jun	**	**	**	NS	**		1.5	1.0	1.3	1.0	0.8	-0.2
Jul	NS	NS	NS	NS	NS	*		-0.5	-0.2	-0.6	-0.7	-1.7
Aug	NS	NS	NS	NS	NS	NS	NS		0.3	-0.04	-0.2	-1.2
Sept	NS	NS	NS	NS	NS	NS	NS	NS		-0.3	-0.5	-1.5
Oct	NS	NS	NS	NS	NS	NS	NS	NS	NS		-0.1	-1.1
Nov	NS	NS	NS	NS	NS	NS	NS	NS	NS	NS		-1.0
Dec	***	***	***	NS	***	NS	**	NS	*	NS	NS	

RED OAK - BRANCH

total NSC

	Jan	Feb	Mar	Apr	May	Jun	Jul	Aug	Sept	Oct	Nov	Dec
Jan		-7.0	-3.3	6.8	0.2	3.6	0.7	-5.3	-3.0	-9.5	-3.7	-0.7
Feb	NS		3.6	13.7	7.2	10.5	7.6	1.6	4.0	-2.5	3.3	6.3
Mar	NS	NS		10.1	3.5	6.9	4.0	-2.0	0.3	-6.1	-0.4	2.6
Apr	NS	***	*		-6.6	-3.2	-6.1	-12.1	-9.7	-16.2	-10.5	-7.4
May	NS	NS	NS	NS		3.4	0.5	-5.5	-3.2	-9.7	-3.9	-0.9
Jun	NS	*	NS	NS	NS		-2.9	-8.9	-6.5	-13.0	-7.3	-4.2
Jul	NS	NS	NS	NS	NS	NS		-6.0	-3.6	-10.1	-4.4	-1.3
Aug	NS	NS	NS	**	NS	NS	NS		2.3	-4.2	1.6	4.6
Sept	NS	NS	NS	*	NS	NS	NS	NS		-6.5	-0.7	2.3
Oct	*	NS	NS	****	*	***	*	NS	NS		5.8	8.8
Nov	NS	NS	NS	*	NS	NS	NS	NS	NS	NS		3.0
Dec	NS	NS	NS	NS	NS	NS	NS	NS	NS	NS	NS	

RED OAK - BRANCH

sugar

	Jan	Feb	Mar	Apr	May	Jun	Jul	Aug	Sept	Oct	Nov	Dec
Jan		-2.8	1.1	7.9	8.9	5.9	6.1	8.1	5.4	6.8	1.3	2.3
Feb	NS		3.9	10.8	11.8	8.8	8.9	11.0	8.3	9.7	4.1	5.2
Mar	NS	NS		6.9	7.9	4.8	5.0	7.0	4.4	5.8	0.2	1.3
Apr	***	****	**		1.0	-2.0	-1.9	0.2	-2.5	-1.1	-6.7	-5.6
May	****	****	***	NS		-3.0	-2.9	-0.8	-3.5	-2.1	-7.7	-6.6
Jun	*	****	NS	NS	NS		0.2	2.2	-0.5	0.9	-4.6	-3.6
Jul	*	****	NS	NS	NS	NS		2.0	-0.6	0.8	-4.8	-3.7
Aug	***	****	**	NS	NS	NS	NS		-2.7	-1.3	-6.8	-5.8
Sept	*	****	NS	NS	NS	NS	NS	NS		1.4	-4.2	-3.1
Oct	**	****	*	NS	NS	NS	NS	NS	NS		-5.6	-4.5
Nov	NS	NS	NS	**	***	NS	NS	**	NS	*		1.1
Dec	NS	NS	NS	*	**	NS	NS	*	NS	NS	NS	

RED OAK - BRANCH

starch

	Jan	Feb	Mar	Apr	May	Jun	Jul	Aug	Sept	Oct	Nov	Dec
Jan		-4.1	-4.4	-1.2	-8.7	-2.3	-5.4	-13.4	-8.4	-16.3	-5.0	-3.0
Feb	NS		-0.3	2.9	-4.6	1.8	-1.3	-9.3	-4.3	-12.2	-0.9	1.1
Mar	NS	NS		3.2	-4.3	2.1	-1.0	-9.0	-4.0	-11.9	-0.6	1.4
Apr	NS	NS	NS		-7.6	-1.2	-4.2	-12.3	-7.2	-15.1	-3.8	-1.9
May	**	NS	NS	*		6.4	3.3	-4.7	0.3	-7.6	3.8	5.7
Jun	NS	NS	NS	NS	NS		-3.1	-11.1	-6.1	-14.0	-2.6	-0.7
Jul	NS	NS	NS	NS	NS	NS		-8.0	-3.0	-10.9	0.4	2.4
Aug	****	**	**	****	NS	****	*		5.0	-2.9	8.5	10.4
Sept	**	NS	NS	NS	NS	NS	NS	NS		-7.9	3.4	5.4
Oct	****	****	****	****	*	****	***	NS	*		11.3	13.3
Nov	NS	NS	NS	NS	NS	NS	NS	**	NS	****		1.9
Dec	NS	NS	NS	NS	NS	NS	NS	***	NS	****	NS	

WHITE ASH - BRANCH												
total NSC												
	Jan	Feb	Mar	Apr	May	Jun	Jul	Aug	Sept	Oct	Nov	Dec
Jan												
Feb												
Mar												
Apr												
May												
Jun												
Jul												
Aug												
Sept												
Oct												
Nov												
Dec												

WHITE ASH - BRANCH												
sugar												
	Jan	Feb	Mar	Apr	May	Jun	Jul	Aug	Sept	Oct	Nov	Dec
Jan		-0.7	2.0	5.0	6.9	5.6	6.6	5.9	5.2	4.3	2.1	2.9
Feb	NS		2.7	5.7	7.6	6.3	7.3	6.6	5.9	5.0	2.8	3.6
Mar	NS	NS		3.0	4.9	3.6	4.6	3.9	3.2	2.3	0.1	0.9
Apr	NS	NS	NS		1.9	0.6	1.6	0.9	0.2	-0.7	-2.9	-2.1
May	***	*	NS	NS		-1.3	-0.3	-1.0	-1.7	-2.6	-4.8	-4.0
Jun	NS	NS	NS	NS	NS		1.0	0.3	-0.4	-1.3	-3.5	-2.7
Jul	NS	*	NS	NS	NS	NS		-0.7	-1.4	-2.3	-4.5	-3.7
Aug	NS	NS	NS	NS	NS	NS	NS		-0.7	-1.6	-3.8	-3.0
Sept	NS	NS	NS	NS	NS	NS	NS	NS		-0.9	-3.1	-2.3
Oct	NS	NS	NS	NS	NS	NS	NS	NS	NS		-2.2	-1.4
Nov	NS	NS	NS	NS	NS	NS	NS	NS	NS	NS		0.8
Dec	NS	NS	NS	NS	NS	NS	NS	NS	NS	NS	NS	

WHITE ASH - BRANCH												
starch												
	Jan	Feb	Mar	Apr	May	Jun	Jul	Aug	Sept	Oct	Nov	Dec
Jan		-2.0	-2.9	0.06	-8.9	-3.8	-3.9	-4.4	-6.7	-8.2	-1.6	-0.7
Feb	NS		-0.9	2.1	-6.9	-1.8	-1.9	-2.4	-4.6	-6.2	0.5	1.3
Mar	NS	NS		2.9	-6.1	-0.9	-1.1	-1.5	-3.8	-5.3	1.3	2.2
Apr	NS	NS	NS		-9.0	-3.9	-4.0	-4.5	-6.7	-8.3	-1.6	-0.7
May	*	NS	NS	*		5.1	5.0	4.5	2.3	0.7	7.3	8.3
Jun	NS	NS	NS	NS	NS		-0.1	-0.6	-2.9	-4.4	2.2	3.1
Jul	NS	NS	NS	NS	NS	NS		-0.5	-2.7	-4.3	2.4	3.2
Aug	NS	NS	NS	NS	NS	NS	NS		-2.3	-3.8	2.8	3.7
Sept	NS	NS	NS	NS	NS	NS	NS	NS		-1.5	5.1	6.0
Oct	*	NS	NS	*	NS	NS	NS	NS	NS		6.6	7.5
Nov	NS	NS	NS	NS	NS	NS	NS	NS	NS	NS		0.9
Dec	NS	NS	NS	NS	*	NS	NS	NS	NS	NS	NS	

RED MAPLE - BRANCH

total NSC

	Jan	Feb	Mar	Apr	May	Jun	Jul	Aug	Sept	Oct	Nov	Dec
Jan		-0.9	2.5	3.3	1.8	-0.4	-1.8	0.4	-4.6	-6.4	-1.8	-0.04
Feb	NS		3.5	4.2	2.7	0.5	-0.9	1.4	-3.7	-5.4	-0.9	0.9
Mar	NS	NS		0.8	-0.7	-3.0	-4.3	-2.1	-7.1	-8.9	-4.4	-2.6
Apr	NS	*	NS		-1.5	-3.7	-5.1	-2.9	-7.9	-9.7	-5.1	-3.3
May	NS	NS	NS	NS		-2.2	-3.6	-1.4	-6.4	-8.2	-3.6	-1.8
Jun	NS	NS	NS	*	NS		-1.4	0.9	-4.2	-5.9	-1.4	0.4
Jul	NS	NS	**	***	NS	NS		2.2	-2.8	-4.6	-0.01	1.8
Aug	NS	NS	NS	NS	NS	NS	NS		-5.0	-6.8	-2.2	-0.5
Sept	**	NS	****	****	****	*	NS	**		-1.8	2.8	4.6
Oct	****	***	****	****	****	***	**	****	NS		4.6	6.3
Nov	NS	NS	**	***	NS	NS	NS	NS	NS	**		1.8
Dec	NS	NS	NS	NS	NS	NS	NS	NS	**	****	NS	

RED MAPLE - BRANCH

sugar

	Jan	Feb	Mar	Apr	May	Jun	Jul	Aug	Sept	Oct	Nov	Dec
Jan		1.4	4.6	3.1	4.2	3.9	3.8	4.6	3.3	3.8	0.8	1.4
Feb	NS		3.2	1.7	2.8	2.5	2.4	3.2	1.9	2.4	-0.6	0.04
Mar	****	****		-1.5	-0.4	-0.7	-0.8	-0.01	-1.3	-0.8	-3.8	-3.1
Apr	****	NS	NS		1.1	0.8	0.7	1.4	0.2	0.6	-2.3	-1.7
May	****	***	NS	NS		-0.3	-0.4	0.3	-0.9	-0.4	-3.4	-2.8
Jun	****	**	NS	NS	NS		-0.1	0.7	-0.6	-0.1	-3.0	-2.5
Jul	****	**	NS	NS	NS	NS		0.8	-0.5	-0.05	-3.0	-2.4
Aug	****	****	NS	NS	NS	NS	NS		-1.3	-0.8	-3.8	-3.1
Sept	****	NS	NS	NS	NS	NS	NS	NS		0.5	-2.5	-1.9
Oct	****	**	NS	NS	NS	NS	NS	NS	NS		-3.0	-2.3
Nov	NS	NS	****	**	****	***	***	****	**	***		0.6
Dec	NS	NS	****	NS	***	**	**	****	NS	**	NS	

RED MAPLE - BRANCH

starch

	Jan	Feb	Mar	Apr	May	Jun	Jul	Aug	Sept	Oct	Nov	Dec
Jan		-2.3	-2.1	0.2	-2.4	-4.4	-5.6	-4.2	-7.9	-10.2	-2.6	-1.5
Feb	NS		0.3	2.5	-0.09	-2.0	-3.3	-1.8	-5.6	-7.8	-0.3	0.8
Mar	NS	NS		2.2	-0.4	-2.3	-3.6	-2.1	-5.8	-8.1	-0.6	0.6
Apr	NS	NS	NS		-2.6	-4.5	-5.8	-4.3	-8.1	-10.3	-2.8	-1.6
May	NS	NS	NS	NS		-1.9	-3.2	-1.7	-5.5	-7.7	-0.2	0.9
Jun	**	NS	NS	**	NS		-1.3	0.2	-3.6	-5.8	1.7	2.9
Jul	****	NS	*	****	NS	NS		1.5	-2.3	-4.5	3.0	4.1
Aug	**	NS	NS	**	NS	NS	NS		-3.7	-6.0	1.5	2.7
Sept	****	****	****	****	****	*	NS	*		-2.3	5.3	6.4
Oct	****	****	****	****	****	****	**	****	NS		7.5	8.7
Nov	NS	NS	NS	NS	NS	NS	NS	NS	****	****		1.2
Dec	NS	NS	NS	NS	NS	NS	**	NS	****	****	NS	

PAPER BIRCH - BRANCH												
total NSC												
	Jan	Feb	Mar	Apr	May	Jun	Jul	Aug	Sept	Oct	Nov	Dec
Jan		-0.8	-1.2	1.0	1.4	1.4	0.5	-2.4	-0.7	-7.3	-3.2	1.1
Feb	NS		-0.3	1.9	2.2	2.3	1.4	-1.6	0.1	-6.5	-2.3	1.9
Mar	NS	NS		2.2	2.5	2.6	1.7	-1.2	0.4	-6.2	-2.0	2.2
Apr	NS	NS	NS		0.3	0.4	-0.5	-3.4	-1.8	-8.3	-4.2	0.04
May	NS	NS	NS	NS		0.07	-0.8	-3.8	-2.1	-8.7	-4.5	-0.3
Jun	NS	NS	NS	NS	NS		-0.9	-3.8	-2.2	-8.8	-4.6	-0.4
Jul	NS	NS	NS	NS	NS	NS		-2.9	-1.3	-7.9	-3.7	0.5
Aug	NS	NS	NS	NS	NS	NS	NS		1.7	-4.9	-0.7	3.5
Sept	NS	NS	NS	NS	NS	NS	NS	NS		-6.6	-2.4	1.8
Oct	*	NS	NS	*	**	**	*	NS	NS		4.2	8.4
Nov	NS	NS	NS	NS	NS	NS	NS	NS	NS	NS		4.2
Dec	NS	NS	NS	NS	NS	NS	NS	NS	NS	*	NS	

PAPER BIRCH - BRANCH												
sugar												
	Jan	Feb	Mar	Apr	May	Jun	Jul	Aug	Sept	Oct	Nov	Dec
Jan		0.3	-0.4	1.1	3.3	3.1	3.8	2.0	2.9	1.9	-0.6	1.2
Feb	NS		-0.8	0.8	3.0	2.8	3.5	1.7	2.6	1.6	-1.0	0.8
Mar	NS	NS		1.6	3.7	3.6	4.3	2.5	3.3	2.4	-0.2	1.6
Apr	NS	NS	NS		2.2	2.0	2.7	0.9	1.8	0.8	-1.8	0.04
May	NS	NS	NS	NS		-0.2	0.5	-1.3	-0.4	-1.4	-4.0	-2.1
Jun	NS	NS	NS	NS	NS		0.7	-1.1	-0.2	-1.2	-3.8	-2.0
Jul	NS	NS	NS	NS	NS	NS		-1.8	-0.9	-1.9	-4.5	-2.7
Aug	NS	NS	NS	NS	NS	NS	NS		0.9	-0.09	-2.7	-0.9
Sept	NS	NS	NS	NS	NS	NS	NS	NS		-1.0	-3.6	-1.7
Oct	NS	NS	NS	NS	NS	NS	NS	NS	NS		-2.6	-0.8
Nov	NS	NS	NS	NS	NS	NS	NS	NS	NS	NS		1.8
Dec	NS	NS	NS	NS	NS	NS	NS	NS	NS	NS	NS	

PAPER BIRCH - BRANCH												
starch												
	Jan	Feb	Mar	Apr	May	Jun	Jul	Aug	Sept	Oct	Nov	Dec
Jan		-1.2	-0.8	-0.1	-2.0	-1.7	-3.3	-4.4	-3.7	-9.3	-2.5	-0.1
Feb	NS		0.4	1.1	-0.8	-0.5	-2.1	-3.3	-2.5	-8.1	-1.3	1.1
Mar	NS	NS		0.7	-1.2	-0.9	-2.5	-3.7	-2.9	-8.5	-1.8	0.6
Apr	NS	NS	NS		-1.9	-1.6	-3.2	-4.3	-3.6	-9.2	-2.4	-0.01
May	NS	NS	NS	NS		0.3	-1.3	-2.5	-1.7	-7.3	-0.6	1.9
Jun	NS	NS	NS	NS	NS		-1.6	-2.7	-1.9	-7.6	-0.8	1.6
Jul	NS	NS	NS	NS	NS	NS		-1.2	-0.4	-6.0	0.8	3.2
Aug	NS	NS	NS	NS	NS	NS	NS		0.8	-4.8	1.9	4.3
Sept	NS	NS	NS	NS	NS	NS	NS	NS		-5.6	1.1	3.5
Oct	****	***	****	****	***	***	**	*	*		6.8	9.2
Nov	NS	NS	NS	NS	NS	NS	NS	NS	NS	**		2.4
Dec	NS	NS	NS	NS	NS	NS	NS	NS	NS	****	NS	

WHITE PINE - BRANCH												
total NSC												
	Jan	Feb	Mar	Apr	May	Jun	Jul	Aug	Sept	Oct	Nov	Dec
Jan		-1.1	-1.5	1.0	-4.5	-2.6	-1.7	-2.2	0.5	-0.02	0.2	0.2
Feb	NS		-0.3	2.1	-3.4	-1.5	-0.6	-1.1	1.6	1.1	1.3	1.3
Mar	NS	NS		2.4	-3.0	-1.2	-0.3	-0.8	2.0	1.4	1.7	1.7
Apr	NS	NS	NS		-5.5	-3.6	-2.7	-3.2	-0.5	-1.0	-0.8	-0.8
May	***	*	NS	****		1.9	2.7	2.3	5.0	4.5	4.7	4.7
Jun	NS	NS	NS	**	NS		0.9	0.4	3.1	2.6	2.8	2.8
Jul	NS	NS	NS	NS	NS	NS		-0.5	2.2	1.7	1.9	1.9
Aug	NS	NS	NS	*	NS	NS	NS		2.7	2.2	2.4	2.4
Sept	NS	NS	NS	NS	****	*	NS	NS		-0.5	-0.3	-0.3
Oct	NS	NS	NS	NS	***	NS	NS	NS	NS		0.2	0.2
Nov	NS	NS	NS	NS	****	NS	NS	NS	NS	NS		-0.01
Dec	NS	NS	NS	NS	****	NS	NS	NS	NS	NS	NS	

WHITE PINE - BRANCH												
sugar												
	Jan	Feb	Mar	Apr	May	Jun	Jul	Aug	Sept	Oct	Nov	Dec
Jan		-0.08	-0.1	2.3	2.6	2.3	2.4	0.7	2.5	1.6	0.9	0.5
Feb	NS		-0.05	2.4	2.6	2.4	2.5	0.8	2.6	1.7	1.0	0.6
Mar	NS	NS		2.4	2.7	2.4	2.5	0.8	2.7	1.8	1.0	0.6
Apr	*	*	*		0.2	0.01	0.06	-1.6	0.2	-0.7	-1.4	-1.8
May	*	**	**	NS		-0.2	-0.2	-1.8	-0.01	-0.9	-1.6	-2.0
Jun	*	*	*	NS	NS		0.05	-1.6	0.2	-0.7	-1.4	-1.8
Jul	*	*	*	NS	NS	NS		-1.7	0.2	-0.7	-1.5	-1.9
Aug	NS	NS	NS	NS	NS	NS	NS		1.8	0.9	0.2	-0.2
Sept	*	*	**	NS	NS	NS	NS	NS		-0.9	-1.6	-2.0
Oct	NS	NS	NS	NS	NS	NS	NS	NS	NS		-0.7	-1.1
Nov	NS	NS	NS	NS	NS	NS	NS	NS	NS	NS		-0.4
Dec	NS	NS	NS	NS	NS	NS	NS	NS	NS	NS	NS	

WHITE PINE - BRANCH												
starch												
	Jan	Feb	Mar	Apr	May	Jun	Jul	Aug	Sept	Oct	Nov	Dec
Jan		-1.0	-1.3	-1.3	-7.0	-4.9	-4.1	-2.9	-2.0	-1.6	-0.7	-0.3
Feb	NS		-0.3	-0.3	-6.0	-3.9	-3.1	-1.9	-1.0	-0.6	0.3	0.7
Mar	NS	NS		0.005	-5.7	-3.6	-2.8	-1.6	-0.7	-0.3	0.6	1.0
Apr	NS	NS	NS		-5.7	-3.6	-2.8	-1.6	-0.7	-0.3	0.6	1.0
May	****	****	****	****		2.1	2.9	4.1	5.0	5.4	6.3	6.7
Jun	****	****	****	****	*		0.8	2.0	2.9	3.3	4.2	4.6
Jul	****	***	**	**	***	NS		1.2	2.1	2.5	3.4	3.8
Aug	***	NS	NS	NS	****	NS	NS		0.9	1.3	2.2	2.6
Sept	NS	NS	NS	NS	****	***	*	NS		0.4	1.3	1.7
Oct	NS	NS	NS	NS	****	****	**	NS	NS		0.9	1.3
Nov	NS	NS	NS	NS	****	****	****	*	NS	NS		0.4
Dec	NS	NS	NS	NS	****	****	****	**	NS	NS	NS	

Table S8 Partitioning of sugar and starch pools among woody organs and sampling months for five temperate tree species at Harvard Forest. The percentage of sugar and starch pools in each organ relative to whole-tree NSC were calculated for each month for each tree. Values represent mean percentages ± 1SE.

	woody biomass (kg)	Percentage (%) of tree NSC by month											
		January		April		June		August		October		December	
		sugar	starch	sugar	starch	sugar	starch	sugar	starch	sugar	starch	sugar	starch
red oak													
root	181 ± 33	20.7 ± 1.8	18.4 ± 3.8	14.3 ± 2.2	28.1 ± 3.9	8.2 ± 1.7	28 ± 4	8.2 ± 0.4	20 ± 2.6	9.7 ± 1.7	14.9 ± 2.4	8.3 ± 1.7	15.8 ± 6.4
stemwood	617 ± 119	27.5 ± 1.8	2.1 ± 0.8	27.9 ± 2.7	11.5 ± 1.1	24.3 ± 3.7	10.2 ± 0.6	20.7 ± 2.8	3.9 ± 0.8	19.9 ± 0.9	7.3 ± 0.3	32.8 ± 4.6	9.9 ± 1.5
branch	203 ± 31	29.6 ± 1.5	1.8 ± 1	13 ± 1.9	5.2 ± 0.6	19.7 ± 2.6	9.7 ± 2	10.6 ± 0.7	36.6 ± 3.8	11.9 ± 0.6	36.4 ± 3.6	24.9 ± 5.2	8.3 ± 1.9
total tree	1001 ± 183	77.7 ± 3.7	22.3 ± 3.7	55.2 ± 4.4	44.8 ± 4.4	52.1 ± 5.4	47.9 ± 5.4	39.5 ± 3.6	60.5 ± 3.6	41.4 ± 1.6	58.6 ± 1.6	66 ± 4.8	34 ± 4.8
white ash													
root	100 ± 24	12.2 ± 0.3	14.3 ± 3	12.2 ± 1.1	19.9 ± 2.3	9.5 ± 0.4	20 ± 2.5	10.3 ± 0.5	19 ± 1.5	10.6 ± 0.5	13.3 ± 1.4	16.7 ± 3.7	11.5 ± 5
stemwood	335 ± 87	25.6 ± 0.9	5.6 ± 2.3	25.1 ± 2.8	9.8 ± 1.3	18.7 ± 1.1	10.1 ± 3.1	20.1 ± 2.3	5.2 ± 1.3	20.7 ± 1.5	6.8 ± 1.1	27.3 ± 5.4	6.8 ± 0.2
branch	120 ± 24	36.7 ± 3.1	5.5 ± 1.4	26.5 ± 0.8	6.5 ± 2.2	21.5 ± 0.7	20.1 ± 4.2	21.2 ± 1.5	24.1 ± 3.3	22.3 ± 2.4	26.3 ± 5.8	29.7 ± 5.4	8 ± 3.2
total tree	554 ± 135	74.5 ± 3.7	25.5 ± 3.7	63.7 ± 2.2	36.3 ± 2.2	49.7 ± 0.2	50.3 ± 0.2	51.6 ± 4	48.4 ± 4	53.6 ± 4.1	46.4 ± 4.1	73.7 ± 6.3	26.3 ± 6.3
red maple													
root	104 ± 15	16.2 ± 1.6	18.6 ± 2.8	12.2 ± 0.4	28.5 ± 2.7	13.4 ± 1.1	31.3 ± 2.1	12.8 ± 0.7	21.2 ± 2	10.5 ± 0.6	17.9 ± 2.6	14.4 ± 2	9.4 ± 3
stemwood	342 ± 53	26.2 ± 0.7	9.7 ± 1.6	20.9 ± 1.2	15.4 ± 1.9	11.2 ± 0.8	12.1 ± 1.1	14.7 ± 1.2	9.7 ± 1.4	14 ± 1.3	7.7 ± 0.9	26 ± 2.8	16.2 ± 1.4
branch	126 ± 15	26.4 ± 1.1	3 ± 1	19.5 ± 1.5	3.5 ± 0.8	12.2 ± 0.7	19.8 ± 2.4	13.9 ± 0.7	27.7 ± 2.6	11.6 ± 0.7	38.3 ± 4	23.9 ± 1.5	10.1 ± 1.1
total tree	572 ± 82	68.8 ± 2.6	31.2 ± 2.6	52.6 ± 2.4	47.4 ± 2.4	36.9 ± 1	63.1 ± 1	41.4 ± 0.8	58.6 ± 0.8	36.1 ± 1.5	63.9 ± 1.5	64.3 ± 2.4	35.7 ± 2.4

paper birch													
root	111 ± 34	16.7 ± 1.7	11.7 ± 1.6	9.7 ± 1.4	17.9 ± 0.7	7.2 ± 2.9	28.1 ± 7.5	8.5 ± 1.5	9.2 ± 1.9	8.9 ± 1	7.1 ± 5	17 ± 2.8	7.7 ± 6.2
stemwood	368 ± 120	25.5 ± 1.9	5.6 ± 0.5	19.2 ± 2.9	10.1 ± 0.6	14.3 ± 2.7	8.6 ± 2.4	15.5 ± 2.7	4.4 ± 1.2	12.4 ± 2.3	6 ± 0.7	29.2 ± 13.3	12.6 ± 2.8
branch	132 ± 33	38.8 ± 5.3	1.7 ± 1	39.9 ± 1.8	3.1 ± 0.7	29.6 ± 6.5	12.3 ± 0.9	33.1 ± 1.7	29.3 ± 1.7	23.1 ± 2.2	42.5 ± 4.3	30 ± 15.2	3.6 ± 1.6
total tree	610 ± 186	80.9 ± 2.9	19.1 ± 2.9	68.8 ± 0.7	31.2 ± 0.7	51 ± 6.3	49 ± 6.3	57.1 ± 2.9	42.9 ± 2.9	44.4 ± 4.5	55.6 ± 4.5	76.1 ± 3.9	23.9 ± 3.9
white pine													
root	139 ± 24	6.3 ± 1.1	9.4 ± 0.5	5 ± 1.2	9.7 ± 1	3.8 ± 2.2	14.1 ± 0.4	0.9 ± 0.2	3.2 ± 1	3.9 ± 1.4	4.1 ± 1	5.2 ± 1.7	3.3 ± 0.8
stemwood	429 ± 74	29.1 ± 1.3	0.2 ± 0.2	23.5 ± 1.8	11.1 ± 1.2	16.3 ± 1	10.1 ± 0.6	20.9 ± 1.6	5.6 ± 0.6	26.5 ± 3.3	6.4 ± 2.3	33 ± 2.1	12.5 ± 1.1
branch	107 ± 17	53.7 ± 1.3	1.2 ± 0.8	38 ± 1.7	12.7 ± 0.9	26.1 ± 1.6	29.6 ± 1.6	46.1 ± 2.5	23.3 ± 3.1	44.5 ± 3.6	14.6 ± 1.5	43 ± 1.6	3 ± 0.3
total tree	675 ± 115	89.1 ± 1.1	10.9 ± 1.1	66.5 ± 1.6	33.5 ± 1.6	46.3 ± 1.8	53.7 ± 1.8	68 ± 2.8	32 ± 2.8	74.9 ± 1.8	25.1 ± 1.8	81.2 ± 1.4	18.8 ± 1.4

Methods S1 NSC concentration measurements and uncertainty

Here we provide a table to summarize how organs were subdivided throughout the year for NSC analyses, note whether sugar and starch concentrations were measured (X), estimated (E), or not applicable (N) for each month, and discuss any discrepancies in terms of these measurements.

	month											
sample	J	F	M	A	M	J	J	A	S	O	N	D
stemwood 0-1cm	X	X	X	X	X	X	X	X	X	X	X	X
stemwood 1-2cm	X	X	X	X	X	X	X	X	X	X	X	X
stemwood 2-3cm	X	X	X	X	X	X	X	X	X	X	X	X
stemwood 3-4cm	X	X	X	X	X	X	X	X	X	X	X	X
stemwood 4-8cm	X	X	X	X	X	X	X	X	X	X	X	X
stemwood 8cm-pith	E	E	E	E	E	E	E	E	E	E	E	E
coarse root 0-1cm	X	N	N	X	N	X	N	X	N	X	N	X
coarse root 1cm-pith	X	N	N	X	N	X	N	X	N	X	N	X
branch multi-year	X	X	X	X	X	X	X	X	X	X	X	X

Coarse roots were sampled in January, April, June, August, October and December 2014, so NSC measurements are only available for these months and the other months are marked as not applicable (N).

NSC analyses were conducted for all organs with the exception of deep in the stem heartwood (stemwood 8cm-pith, marked (E)). We measured sugar and starch concentrations in stemwood 8cm-pith for all 24 trees in January and July 2014 and conducted paired t-tests to see if sugar concentrations differed between months for each species (QURU, P=0.03, FRAM, P=0.30, ACRU, P=0.02, BEPA, P=0.20, PIST, P=0.12). Starch concentrations for January and July were 0 mg/g. Since the majority of species did not have sugar concentrations that significantly differed between January and July, average sugar and starch concentrations for January and July were established for each tree and those values were applied for stemwood 8cm-pith (heartwood) for the entire year, including January and July.

Multi-year (3-5 year) branchwood was obtained from sunlit branches for NSC analysis. Due to poor weather conditions, paper birch tree BEPA110 could not be accessed with the bucket lift to collect a branch sample in February, March, and April. Sugar and starch concentrations were similar between the 3 paper birch trees in this study in flanking months (January and May), so the average sugar and starch concentrations for BEPA113 and BEPA106 in February, March, and April were used as the concentrations for BEPA110 for the 3 months when a branch could not be collected.

Additionally, absorbance values to calculate NSC concentrations were measured either by plate reader or spectrometer. We used a plate reader from November 2014 to November 2016 and then switched to a spectrometer for the remainder of the study to increase efficiency. Demonstrating with a subset of samples (n=24), we measured NSC concentrations using both instruments and conducted a paired t-test to determine that calculated sugar (P=0.25) and starch (P=0.53) concentrations do not significantly differ between measurement instruments.
We also included at least one internal laboratory standard (red oak stemwood, Harvard Forest, Petersham, MA, USA) per NSC analysis to assess uncertainty in NSC concentrations. Measured

sugar and starch concentrations for the standard were within ±10% of the mean (sugar, 35.9 ± 3.8 mg/g; starch, 24.5 ± 2.1 mg/g).

Methods S2 Allometric scaling from NSC concentrations to whole-tree pools

Using Jenkins *et al*. (2004), we calculated total aboveground biomass, B (kg), as a function of diameter at breast height, DBH (cm):

$$B = \exp(\beta_0 + \beta_1 \ln \mathrm{DBH})$$

From Table 1 in Jenkins *et al*. (2004), we used the following parameters for our five species to calculate B for each:

species in our study	species group, Table 1	β_0	β_1
red oak	hardwood: maple/oak/hickory/beech	-2.0127	2.4342
white ash	mixed hardwood	-2.4800	2.4835
red maple	hardwood: maple/birch	-1.9123	2.3651
paper birch	hardwood: maple/birch	-1.9123	2.3651
white pine	pine	-2.5356	2.4349

B includes foliage, branches, stemwood, and bark. We then calculated the fraction (f) of B corresponding to foliage, stemwood, and bark, using values of α_0 and α_1 from their Table 2, as:

$$f = \exp(\alpha_0 + (\alpha_1 / \mathrm{DBH}))$$

We used hardwood parameters for red oak, white ash, red maple, and paper birch, and softwood parameters for white pine. For example, for red oak we used α_0= - 0.3065 and α_1= - 5.424 to determine the stemwood fraction. Taking a single red oak tree that we sampled, say QURU104 with DBH=43cm, we estimated the stemwood fraction to be 0.649. We then completed these calculations for all trees and all fractions, determining the branch fraction by difference:

$$f_{\mathrm{branch}} = 1 - f_{\mathrm{foliage}} - f_{\mathrm{stemwood}} - f_{\mathrm{bark}}$$

A similar scaling approach is used in Jenkins *et al*. (2004) to estimate coarse root biomass as a function of B. For QURU104, we calculated $f_{\mathrm{coarse\ roots}} = 0.188$. Our estimates are consistent with a root-to-shoot ratio of 1:5 in temperate forest trees (Richardson *et al.* 2015). It is important to note that Jenkins *et al.* (2004) does not provide equations or coefficients for distinguishing between coarse and fine roots. Thus, only coarse roots were sampled and coarse root biomass was estimated in this study. Richardson *et al*. (2015) provides estimates for fine roots under different biomass scenarios in red oak and white pine.

Based on f for each component, we determined the woody biomass of each component, bm (kg), as:

$$bm = B * f$$

We would then pair sugar, starch, and total NSC concentrations with the estimate of *bm*, multiply together, and then sum over components to determine the whole-tree pools.

However, since we subdivided organs for NSC analyses, we determined *f* and *bm* for each subdivision, and then paired them with sugar and starch concentrations measured for each subdivision. DBH measurements allowed us to partition biomass into subdivisions in the stem. Since we did not have coarse root diameter measurements, we partitioned biomass to root subdivisions by assuming that the ratio of (0-1cm root/ total root) was the same as (0-1cm stemwood/total stemwood). We summed over the following components to determine whole-tree total NSC, sugar, and starch pools in this study: root 0-1cm root, root 1cm-pith, stemwood 0-1cm, stemwood 1-2cm, stemwood 2-3cm, stemwood 3-4cm, stemwood 4-8cm, stemwood 8cm-pith, and multi-year (3-5 year) branchwood.

The average sapwood thickness of our trees was approximately 6cm, and the innermost stemwood subdivisions were 4-8cm and 8cm-pith. As noted in Methods S1, for each tree, NSC concentrations in 8cm-pith stemwood were measured in January and July, these concentrations were averaged together, and the average concentration was applied to all months of the year including January and July. Thus, inclusion of heartwood (8cm-pith) did not impact the seasonal dynamics of stemwood pools. A larger proportion of stemwood heartwood was included for individual trees with sapwood less than 4cm thick.

Methods S3 Estimation of foliar NSC pools

While the whole-tree total NSC pools we provide comprise coarse roots, stemwood, and branches, they do not consider foliage. Average $f_{foliage}$ for our 24 trees was 0.03. Foliage from each tree was collected in July 2014, and sugars and starch were measured to determine the size of foliar total NSC, sugar, and starch pools. Foliage added very little to the whole-tree total NSC pool in July, approximately 1.06 ± 0.54 kg of NSCs.

species	tree	month	foliar sugar pool (kg)	foliar starch pool (kg)	foliar total NSC pool (kg)
ACRU	102	July	0.54	0.25	0.80
ACRU	103	July	0.19	0.09	0.28
ACRU	105	July	0.20	0.16	0.36
ACRU	112	July	0.41	0.34	0.74
ACRU	120	July	0.34	0.32	0.66
ACRU	124	July	0.32	0.36	0.68
BEPA	106	July	0.62	0.12	0.74
BEPA	110	July	1.30	0.83	2.13
BEPA	113	July	0.72	0.23	0.95
FRAM	101	July	0.70	0.35	1.04
FRAM	108	July	0.69	0.46	1.14
FRAM	123	July	0.40	0.15	0.55
PIST	111	July	0.56	0.22	0.78
PIST	114	July	0.89	0.60	1.50
PIST	116	July	0.72	0.17	0.89
PIST	119	July	1.66	0.30	1.96
PIST	121	July	1.18	0.11	1.29

PIST	122	July	1.62	0.47	2.10
QURU	104	July	1.45	0.19	1.64
QURU	107	July	1.11	0.33	1.44
QURU	109	July	1.42	0.24	1.65
QURU	115	July	0.38	0.21	0.60
QURU	117	July	0.39	0.13	0.53
QURU	118	July	0.71	0.33	1.04

Additionally, to examine how the size of whole-tree total NSC pools changes when foliage is considered over several months, we chose a representative deciduous broadleaf tree (red oak QURU109) and evergreen conifer (white pine PIST 119) and measured sugar and starch concentrations in foliage over time. Current-year foliage from QURU109 was analyzed throughout the 2014 growing season spanning May through October, and previous-year foliage from PIST119 was analyzed throughout the entire year. We used these concentrations to scale-up to the whole-tree level and to compare whole-tree total NSC pools with and without foliage for QURU109 and PIST119 over several months. Only months when roots were also sampled and the whole-tree pool was complete were included and reported below. Across these months, including foliage in our calculations increased the whole-tree total NSC pool by approximately 3.6 ± 1.6 kg of total NSCs.

QURU109

month	whole-tree total NSC pool (kg): root, stem, branch	whole-tree total NSC pool (kg): root, stem, branch, foliage
June	35.9	38.8
August	46.5	49.7
October	74.2	76.7

PIST119

month	whole-tree total NSC pool (kg): root, stem, branch	whole-tree total NSC pool (kg): root, stem, branch, foliage
January	21.4	23.6
April	21.6	25.6
June	26.7	33.7
August	19.2	24.2
October	13.4	17.0
December	23.6	25.4

Methods S4 Estimation of ecosystem-level NSC storage

The 14 species used to estimate ecosystem-level total NSC storage included *Acer rubrum* (red maple), *Betula alleghaniensis* (yellow birch), *Betula lenta* (black birch), *Betula papyrifera* (paper birch), *Betula populifolia* (grey birch), *Fagus grandifolia* (American beech), *Fraxinus americana* (white ash), *Picea glauca* (white spruce), *Pinus resinosa* (red pine), *Pinus strobus* (white pine), *Prunus serotina*(black cherry), *Quercus rubra* (northern red oak), *Quercus velutina* (black oak), and *Tsuga canadensis* (eastern hemlock)

Measured total NSC concentrations from our five study species were used as estimates for these 14 species. For example, total NSC concentrations from paper birch (*Betula papyrifera*) were used for *Betula populifolia* and *Betula lenta*. For species like *Fagus grandifolia* that did not have a comparable species in our study, we estimated the total NSC concentrations based on wood anatomy; *Fagus grandifolia* is diffuse-porous, so the mean total NSC concentrations from diffuse-porous species in our study (red maple and paper birch) were used. All foliar NSC estimates were based on NSC concentrations from red oak and white pine.

References

Jenkins JC, Chojnacky DC, Heath LS, Birdsey RA. 2004. Comprehensive database of diameter-based biomass regressions for North American tree species. USDA Forest Service. Northeastern Research Station, Newtown Square, PA, USA.

Richardson AD, Carbone MS, Huggett BA, Furze ME, Czimczik CI, Walker JC, Xu X, Schaberg PG, Murakami P. **2015**. Distribution and mixing of old and new nonstructural carbon in two temperate trees. *New Phytologist* **206**: 590–597.
